# A multilevel, multi-mode framework for standardization in digital B2B platform eco-systems in international cargo transportation—A multiple case study

**DOI:** 10.1007/s12525-022-00551-1

**Published:** 2022-06-09

**Authors:** Ruben Tessmann, Ralf Elbert

**Affiliations:** Chair of Management and Logistics, Technical University of Darmstadt, Hochschulstraße 1, 64289 Darmstadt, Germany

**Keywords:** Standardization, Digital platforms, Eco-systems, Case Study, Transportation, Innovation diffusion, L15, O32, L86, L91, L32

## Abstract

Standardization is gaining importance for digital platform eco-systems as they try to balance both stability and flexibility of their core as well as their boundary resources. Our knowledge on which factors influence standardization processes in business-to-business (B2B) focused digital platform eco-systems remains limited. Using three B2B platform eco-systems from the cargo transportation and seaport eco-system context, we apply a multiple case study to investigate which factors affect the standardization mode and the standard adoption. Based on 19 interviews and a systematic coding procedure, we identified 24 factors and cluster them into four overarching themes. We show the relatedness between standardization and innovation research, identify standard characteristics as the most important theme of factors and show that standardization has to be seen as a dynamic and interconnected process, as some factors are interdependent but the controllability varies between factors. Lastly, implications of these results and future research directions are discussed.

**Supplementary Information:**

The online version contains supplementary material available at 10.1007/s12525-022-00551-1.

## Introduction

Standardization aims to resolve situations where actors generally prefer a common solution to a problem, but have not yet agreed which option to choose and it therefore limits the number of solutions when using many different options simultaneously would be ineffective and inefficient (Wiegmann et al., 2017). We apply a broad understanding of the term “standard”, as we explicitly do not want to limit our research to a certain type of standard, such as accredited standards which are defined by an SDO such as ISO. Therefore, we also explicitly, but not exclusively include industry specifications and de-facto standards and summarize them under the term “standards” (NPES, 2010, Chapter 2). Standards affect many aspects of the economic activity of singular businesses such as R&D or production and also influence the dynamics in entire industries or economies by affecting market penetration, power distribution and more. They therefore have a significant collective effect on innovation, productivity, and market structure (Tassey, 2000). Accordingly, a better understanding of standardization processes and mechanisms can help to find better standards in the sense of maximizing economic welfare and efficiency without getting “trapped” with an obsolete or inferior standard (Farrell and Saloner 1985). Additionally, reaching a standard in a shorter time with less resources, i.e., more efficiently, can be supported by deeper, context-specific knowledge (Wiegmann, 2019).

To have a clear common understanding of the different perspectives and related terms we will use in this paper, we want to briefly demarcate the three perspectives here. Firstly, we distinguish the stages of standardization, i.e., between pre-development, development, and maintenance of a standard. During the pre-development stage, no actions are taken in order to define a standard (yet), while during the standard development stage, the standard is not yet defined in its entirety, but one or more stakeholders are working on it. Lastly, during standard maintenance, a previously defined standard is kept “up to date”. Secondly, from a different viewing angle, it is relevant where, how and by whom a standard is developed or maintained, which is distinguished by the standard activation mode or just (ideal-typical) mode of standardization^1^ (Wiegmann et al., 2017). A third perspective can be chosen, which does not focus on the definition or development of the standard but on its usage. Here, we borrow from the well-established adoption and diffusion literature body (e.g., (Jeyaraj et al., 2006; Robey et al., 2008; Rogers, 1995)) which aims to explain the factors influencing the adoption, i.e., active usage, of an innovation or as in our case a standard by an individual, an organization or a group of such. The diffusion is then defined as the time-related development of adoption decisions, i.e., how many adopters an innovation has at a certain point in time (Rogers, 1995). Accordingly, when we speak of standard diffusion, we mean the rapidity and extent to which a standard is adopted.

The dominant view of researchers on standardization is focusing on the activation or just mode of standardization (Wiegmann et al., 2017). Wiegmann et al. (2017) distinguish the market-based, committee-based and government-based modes. Unfortunately, our knowledge of how standards are developed, adopted and maintained across multiple modes of standardization by a variety of stakeholders and what influences these processes remain limited (Shin et al., 2015; Wiegmann et al., 2017). Commonly, the three ideal-typical modes of standardization are treated as mutually exclusive to each other and only a limited number of studies and reports exist that examine, explicitly or implicitly, the peculiarities of multi-mode standardizations (e.g., (Bakker et al., 2015; Lu et al., 2016; Meyer, 2012)), i.e., standardization processes that are pursued through more than one mode (e.g., combined market-based and committee-based standardization). We refer to the modes as activation modes, as one of the key distinguishing factors between the ideal-typical modes of Wiegmann et al. (2017) is which (set of) stakeholders activate the respective mode, e.g., a committee in the committee-based mode. Wiegmann et al. (2017) point out the relevance of the institutional and technological context in multi-mode standardization.

The development and implementation of various types of standards has a long and eclectic history in the international cargo transportation context (IMO, 2013). In an industry where many actors work in close relation to transport billions of tons of cargo, various standards regarding information, equipment, processes and more, are necessary to enable an effective and efficient exchange amongst all involved stakeholders (cf. exemplarily (Egyedi & Spirco, 2011; Meyer, 2012)). With the entrance of new market participants such as ICT companies, banks or insurances, the choice and combination of modes of standardization may change (Wiegmann et al., 2017).

Globally, many industries are converging and previously unrelated stakeholders get connected through emerging platforms (van de Kaa et al., 2015). Here, standardization efforts, especially of boundary resources, i.e., the technical, organizational, social and other links provided by a digital platform (core) to connect with its respective eco-system (de Reuver et al., 2018; Hein et al., 2020), are gaining in importance drastically, in order to find a common ground for cooperation and coopetition on the platform (Hein et al., 2019; Rodon et al., 2007, 2008). Both platform and standardization research show a high case-by-case variation, which makes contextualization highly relevant, as it allows for a better comparability of results between studies and therefore also facilitates a deeper understanding (de Reuver et al., 2018; Wiegmann et al., 2017). For example, the focus on activation modes often leads to standards merely being treated as complex black-boxes (Egyedi & Spirco, 2011; Tassey, 2000) which can hamper the comparability of results.

For our study, we choose the port eco-system context which is particularly interesting, as here, digital platforms connect competitive, international business-to-business (B2B) cargo transportation networks (Wallbach et al., 2019) with deeply involved governmental stakeholders, such as port authorities or customs which exert strong influence on the platform and its members (Chandra & van Hillegersberg, 2018; Rodon et al., 2008). Port Community Systems (PCS) are one type of such platforms that focus on a singular seaport and its community (Moros-Daza et al., 2020). We study three platform eco-systems that are set in the same context, but are explicitly no singular PCS, as they work on overcoming local PCS’ (geographic) limitations, by connecting multiple PCS internationally and by providing platforms for the entire (intermodal) port hinterland and inland transport. To find out more about which factors influence the standard activation modes in B2B platform eco-systems, we ask:

- **RQ 1:** Which factors influence the choice and combination of standard activation modes in B2B platform eco- systems?

The knowledge on factors that influence standard adoption is unequally distributed between the standardization modes. For committee-based standardization efforts, where the coordination takes place during standard development, extant literature on adoption factors is in its infancy (Wiegmann et al., 2017). When coordination takes place during the diffusion process (market-based), the adoption of standards and (technological) innovations is highly similar, as a company’s innovation is often turned – through a battle of dominance – into a standard (van de Kaa et al., 2011) and accordingly the literature body is richer (Shin et al., 2015; Techatassanasoontorn & Suo, 2011). Even for market-based standardization, insights for B2B platform eco- systems are scarce, though. Digital platform research, which mostly focuses on consumer-oriented platforms, still lacks knowledge on reasons for success or failure of digital platforms, including the standards they develop, adopt and enforce (de Reuver et al., 2018). As insights from consumer-oriented digital platforms can only be transferred to their B2B counterparts with caution (Wallbach et al., 2019), contextualized insights in the B2B context are even more necessary (Hein et al., 2019; Loux et al., 2020). Accordingly, we ask:

- **RQ 2:** Which factors influence the adoption of standards in B2B platform eco-systems?

Methodologically, we follow a multiple case study comprising three B2B platforms in the context of port eco- systems (Yin, 2017). We address recent calls for more contextualized insights on multi-mode standardization (Wiegmann et al., 2017) and introduce a multi-level framework of contextual factors influencing the mode choice and adoption of standards. Similar multi-level research approaches gained popularity amongst the platform and inter-organizational information systems (IOIS) adoption and assimilation research community as a means to address the complexity and better contextualize studies (de Reuver et al., 2018; Kurnia et al., 2019; Zhang & Gable, 2017). In contrast to the more specific multi-level perspective approaches known from transition studies (e.g., (Geels, 2002; Van Bree et al., 2010; Walrave et al., 2018)), a broader multi-level research approach emerged in our context, i.e., factors could be grouped into different levels which supports the contextualization (Kurnia et al., 2019; Molina-Azorín et al., 2019). Accordingly, multi-level research has been called for in the context of inter-organizational system adoption research by Kurnia et al. (2019) and should, due to similarities of standard and innovation adoption (Shin et al., 2015), be applied in standards research also. Additionally, we contribute towards a deeper understanding of sectoral differences in barriers and facilitators of digital platform assimilation (de Reuver et al., 2018), as we choose the port eco-system context where standards are becoming increasingly important due to multiple industries converging on digital platforms (van de Kaa et al., 2015; Wiegmann et al., 2017). Lastly, we add to the limited body of literature on platform eco-systems in B2B contexts (Hein et al., 2019; Loux et al., 2020), with the specialty of strong governmental influence which is still understudied (Bivona & Cosenz, 2021; de Reuver et al., 2018; Täuscher & Laudien, 2018).

The remainder of this paper unfolds as follows: First, we give an overview of the theoretical background of platform eco-systems and give an overview of the contemporary standardization literature body. Then we present the methodology and the research cases which is followed by the results of our multiple case study. Finally, we discuss the results, present theoretical and practical implications and show avenues for future research.

## Theoretical background

### Platform eco-systems

As eco-systems have received increasing research interest lately, a shared understanding of what a platform- based eco-system is, is slowly emerging (Bogers et al., 2019; Hein et al., 2019). Accordingly, we present our understanding of what characterizes a (digital) platform and its respective eco-system. In accordance with de Reuver et al. (2018)’s definitions of digital platforms and eco-systems, we choose a sociotechnical view on both concepts, as we are interested in the technical elements and the interdependent organizations, associated organizational processes and especially standards (de Reuver et al., 2018, p. 127).

Multi-sided platforms (MSP) are a type of platform that mediate between different distinct groups of stakeholders (Boudreau & Hagiu, 2009). Platforms are commonly seen as a stable core and a variable periphery from both a technical and a socio-technical perspective (de Reuver et al., 2018). As platforms bring together multiple user groups, they create the so-called network externalities. These network externalities are positive or negative effects on a stakeholder that arise from other platform participants either from the same or another side (cf. Evans & Schmalensee, 2016; Parker et al., 2016 for the distinction between positive, negative, same-sided and cross-sided network effects). These network effects arise from supermodular complementarities of two products, services, assets or activities which means that, for example, more of a product A makes a service B more valuable (Eaton et al., 2015; Jacobides et al., 2018).

All stakeholders together are referred to as the eco-system members (Bogers et al., 2019). In order to orchestrate this complex network of complementors, partners and other members, the platform sponsor needs to identify, implement and enforce dedicated governance arrangements that facilitate value-creating mechanisms (Bogers et al., 2019; de Reuver et al., 2018; Hein et al., 2020). This creates a paradox of control, as a balance between centralized and distributed control has to be found permanently (Tilson et al., 2012; Wareham et al., 2014) and a paradox of change, as the digital platform needs to be stable in order to provide a foundation for further stakeholders but also flexible enough to allow for growth (de Reuver et al., 2018; Tilson et al., 2010).

The link between the digital platform (core) and its respective eco-system is referred to as the boundary which is characterized by its boundary resources (de Reuver et al., 2018; Hein et al., 2020). Digital affordances offered to the eco-system through boundary resources are crucial for platform eco-systems as they fuel generativity (de Reuver et al., 2018; Hein et al., 2020; Henfridsson & Bygstad, 2013). Digital affordances refer to “*what an individual or organization with a particular purpose can do with a technology*” (Majchrzak & Markus, 2012) and generativity is the capacity to produce unprompted changes driven by varied and uncoordinated stakeholders (Hein et al., 2020). Through modularization, this implies opportunities for distributed development and recombinant innovation (Clark & Baldwin, 2002). The provision of (technical) boundary resources such as interfaces or toolkits can act as governance mechanisms (Hein et al., 2020).

Bogers et al. (2019) argue that MSP often are transactional instead of relational and therefore are not structured to allow for or enable interdependence, i.e., cooperation, competition or coopetition between the stakeholders of a platform, which would be essential for a joint value creation. Here, Bogers et al. (2019) show a narrower understanding of an eco-system compared to Hein et al. (2020), as they solely see eco-systems with an innovation based value-creating mechanism as such. Contrary to this, Hein et al. (2020) explicitly include platforms that create value through transactions, as long as they follow an underlying supermodular complementarity logic (see innovation eco-system vs. transaction eco-system). For the remainder of this paper we follow the wider view of Hein et al. (2020), as a clear distinction between transaction and innovation eco- systems is sometimes difficult and transaction focused digital platforms can develop into innovation focused ones over time (Elbert & Tessmann, 2021).

### Standardization and innovation

Standardization aims to resolve situations where actors generally prefer a common solution to a problem, but have not yet agreed which option to choose and it therefore limits the number of solutions when using many different options simultaneously would be ineffective and inefficient (Wiegmann et al., 2017). As Wiegmann et al. (2017) point out, standardization and the respective literature body are in certain ways inconsistent, as both fail to follow a “standardized” way of describing and following standards and standardization processes.

Platform eco-systems including their respective boundary resources and standardization efforts have multiple links. First, standardization is an important pillar for a platform’s core. Without existing and evolving standards, the development and upkeep of a (digital) platform’s core would be much costlier. Take, for example, the security of critical and essential data at rest. To ensure such security, a platform could develop and offer its own, proprietary encryption solution instead of using the Advanced Encryption Standard (AES) established by the U.S. National Institute of Standards and Technology in 2001 (NIST, 2001). Not utilizing the standard would mean not only a significant amount of work to develop such a proprietary solution but would also require a valid justification towards the platform’s stakeholders why it is essential to deviate from the standard and to proof the reliability and trustworthiness of the proprietary solution. Accordingly, standardization is, quite necessarily, an essential part of almost all (digital) platforms. Second, standardization can be essential for platform sponsors to find a balance between stability and flexibility especially in the context of boundary resources (Eaton et al., 2015; Hein et al., 2020; Tilson et al., 2012). For example, different types of technical or organizational standards can have a major effect on the development of a platform and its stakeholders. If an interface standard that connects the core of the platform with its users, is defined too closed-off, it might not allow for novel and innovative solutions which ultimately can hinder the growth and prosperity of the entire platform. If the interface standard is defined too open though, the coherent exchange of information can be impeded. Nevertheless, specific, standardization-focused insights are rare, with the exception of Hein et al. (2019), who – amongst other things – investigate the leveraging of boundary resources through a standardization process in a B2B platform context. Lastly, standardization and platform development are closely interwoven. As successful platforms can reach a large number of stakeholders in a short time, they can be a major player in market-based, which is also referred to as “de-facto” standardization where a standard’s acceptance is based on its common usage rather than the support of a standardization institution. On the other hand, the creation and enforcement of new standards, for example regarding data privacy, can also influence the platform and how it is doing business.

**Fig. 1:**
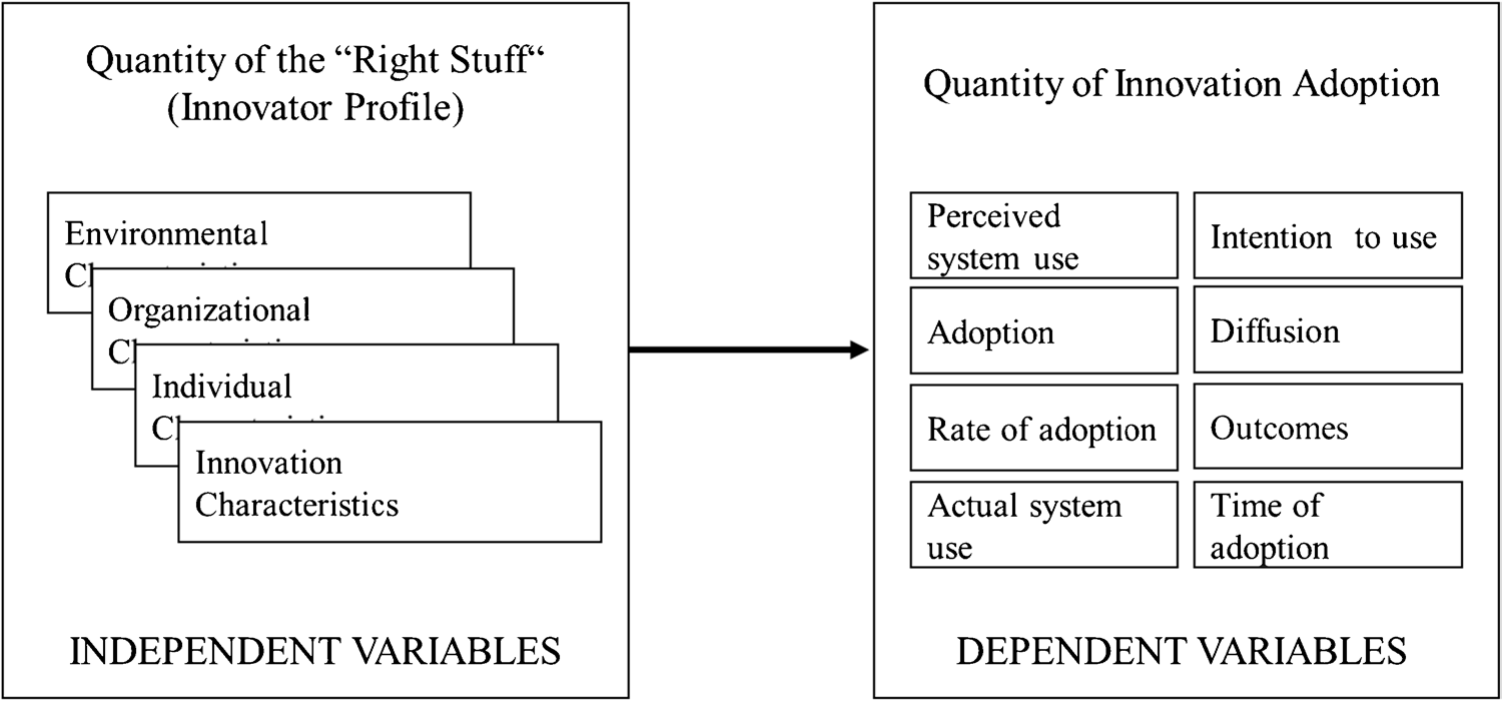
The dominant paradigm for IT innovation adoption. Adopted from (Fichman, 2004; Jeyaraj et al., 2006)

Extant literature commonly also sees a close connection between standardization efforts and the adoption of (IS) innovations (Wright et al., 2012; Zoo et al., 2017). Standardization is seen as a facilitating factor or barrier for innovations (Shin et al., 2015; Wright et al., 2012), a facilitator of trust (Viardot, 2017) or as a subsequent step to an innovation (Acemoglu et al., 2012). Due to the strong focus of extant standardization literature on coordination mechanisms, the study of factors influencing the adoption of committee-based standards, where the coordination takes place during development, is in its infancy (Wiegmann et al., 2017). When looking at market- based standardization, standards and innovations might be viewed as one and the same thing, as a company’s or a consortium’s innovation (e.g. the VHS system for video recording (Cusumano et al., 1992)) could turn into a standard through fast and significant adoption and diffusion (van de Kaa et al., 2011; Wiegmann et al., 2017)). Accordingly, standard adoption literature sometimes applies a paradigm that is similar to that of innovation adoption (Adebesin et al., 2013; van de Kaa et al., 2011; Wang et al., 2016). This dominant research paradigm (see Fig. 1), first identified by Fichman (2004), assumes that certain independent variables, also referred to as “facilitators and barriers” or “characteristics”, influence dependent variables that measure the quantity of innovation adoption (Jeyaraj et al., 2006). By analogy with innovation characteristics which are relevant in IS innovation adoption research (Jeyaraj et al., 2006), Wiegmann et al., (2017, Chapter 3.3), for example, assume that standard development is dependent on standard characteristics.

The predominant view on standardization treats modes of standardization as mutually exclusive to each other, although some recent studies on converging industries and new governmental approaches prompt the conclusion that a combination of these modes is used in certain cases (Meyer, 2012; Wiegmann et al., 2017). In their literature review Wiegmann et al. (2017) also collect several factors that potentially influence the combination of modes, viz. the standardization “culture” (How has standardization been “done” in the past? – see appendix), the availability of resources and knowledge, the institutional or industry context (coordinated vs. liberal market economy) as well as the technological context (uncertainty and complexity of the underlying technology). As the standardization “culture”, but also the institutional and technological context can change over time, Wiegmann et al. (2017) suggest that a dynamic view on standardization can be beneficial.

Lastly, some IS innovation studies (Keceli et al., 2008; Wallbach et al., 2019) suggest that the factors that are commonly referred to as “independent variables” in the dominant paradigm of innovation adoption, actually show interdependences and should accordingly not be referred to as independent (Fichman, 2004). This is based on insights from technology acceptance and innovation adoption literature, where newer models show interdependencies and influences of multiple factors on each other (Venkatesh et al., 2016). Furthermore, digital platform eco-systems have to be seen as complex socio-technical systems and innovations (de Reuver et al., 2018) which should even increases the potential of interdependencies between variables.

## Methodology

### Multiple case study design

We follow a multiple case research method which is particularly suitable if one aims to capture and describe the complexity of novel phenomena (Yin, 2017). The multiple case study covers the standardization efforts of three B2B platforms eco-systems that illustrate factors influencing the choice of one or multiple standardization modes as well as the adoption of standards. A cross-case analysis allows us to draw more robust conclusions on standardization practices by contrasting and replicating our findings from individual cases (Yin, 2017). We shortly elaborate on why the usage of a case study design is appropriate for our context (Benbasat et al., 1987). Firstly, it is important to observe the utilization and development of standardization practices in B2B platforms in a context-dependent environment (de Reuver et al., 2018; Wiegmann et al., 2017). Secondly, the fast and overwhelming success of consumer-oriented platform eco-systems shows the significance and relevance of the research topic (Murphy et al., 2021; Reck, 2021). Additionally, while these consumer-oriented platform eco- systems already thrive, their B2B counterparts are still in their infancy (Riemensperger & Falk, 2020). The phenomenon enjoys a theoretical base, building on standardization and innovation adoption literature but focusing on the context of B2B platforms that has received little attention so far (Hein et al., 2019). Lastly, the standardization activities studied are grounded in a real situation described by the case studies (Siggelkow, 2007).

To increase construct validity (Yin, 2017), we gathered both primary and secondary (provided documents, archival records) data. The primary data for each case was gathered through semi-structured interviews which had to be held through an online conferencing system due to the active Covid-19 social distancing measures in Europe during the first half of 2021. Neither control nor manipulation of the subjects or events took place, as the case studies describe the phenomenon in the view of a neutral observer. We chose semi-structured interviews as they provide room for improvisation and exploration of the underlying phenomenon.

**Fig. 2:**
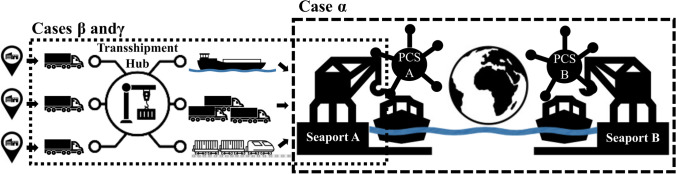
Overview and geographical scope of cases

From a methodological viewpoint, we apply a replication logic, i.e., choose our cases based on relevant differences and accordingly we do not expect highly subtle differences in the results of our cases. Therefore, three cases can be considered sufficient in order to achieve high external validity (Yin, 2017). We choose two case-selection criteria, viz. the platform type in the chosen context of international cargo transportation chains and the success or failure of said platforms. For a better contextualization, we chose our cases to be digital multi- sided B2B platforms with their respective eco-systems that cover one or multiple steps of an (international) supply or transportation chain (see Fig. 2). We choose this context, as, on the one hand, digitalization and the diffusion of multi-sided platforms in the transportation sector are still in the fledging phase (Wallbach et al., 2019) and, on the other hand, this industry has a long history in (international) standardization which makes it an interesting context to study. From a theoretical and conceptual standpoint, we identified three general platform types along supply or transportation chains that are all in the context of port eco-systems, viz. PCS, digital freight exchanges and international exchange platforms. We did not want to focus on PCS platforms, as they already received abundant research attention and are locally-bounded to a singular port and therefore only provide highly localized insights (Moros-Daza et al., 2020). Due to the strong localization, PCS commonly do not cover transportation processes beyond a port’s borders comprehensively and therefore, specialized platforms for these parts of supply and transport chains emerge (Elbert & Gleser, 2019; Jain et al., 2020), viz. what we call digital freight exchanges. We choose two cases (*Beta* and *Gamma*) from this digital freight exchange type that match supply and demand in the transport sector and additionally provide an information exchange. *Alpha* connects many local PCS platforms with their variety of private businesses from multiple sectors including banks, for example, and governmental stakeholders on an international level, so the eco-system is rather broad. Secondly, we distinguish based on the success trajectory of the potential platforms to study. As the success of a platform is regularly not defined precisely in extant literature (e.g., (Casey & Töyli, 2012; Zhao et al., 2020)), we choose to measure it in continuous member growth, i.e., the ongoing diffusion of its services. Unsuccessful or failing cases have not been considered regularly in extant research (Wallbach et al., 2019), which bears the risk of biased results (Jeyaraj et al., 2006), so we want to explicitly include also unsuccessful cases to see if this has any influence on the standardization efforts. In this dimension, *Beta* is a thriving platform with a strong growth rate while *Gamma* can be considered a failed case, as its operation has been terminated. We initially wanted to apply a similar logic to the third platform type, i.e., platforms focusing on international data-exchange and transactions, but could not identify a failed case that had a similar scope to *Alpha*. International digital freight exchanges address a smaller eco-system than *Alpha*, as they focus on the organization of cargo transportation and therefore focus solely on businesses from the transportation sector (Jain et al., 2020).

Case *Alpha* is a platform in an early stage that aims to connect a range of PCS operators which have decades of experience in developing, running and maintaining platform eco-systems and also a long history of developing, implementing and enforcing (international) standards with high governmental influence. While cases *Beta* and *Gamma* are also in early stages, their platform sponsors are small start-ups that aim to revolutionize the intermodal in- and hinterland container transport in Europe (not solely the EU) and therefore neither have a similar level of prior experience with platform eco-systems nor with standard development and adoption. All cases satisfy the conditions of platform eco-systems as they bring together multiple user groups, thereby creating network externalities, and utilize governance provisions as collective arrangements between their members.

They are emerging as transaction-focused platform eco-systems, but can transition into innovation eco-systems, as the exchanged data can be utilized to offer new services and products. A short description of each case can be found in Table 1 below and we included a more detailed description and classification in the appendix (Classification of the cases as digital platform eco-systems).

**Table 1:** Overview of cases based on the case selection criteria

	Contextual perspective – Chosen platform types along an (international) transport chain	
	International exchange platforms	Digital (localized) freight exchanges
Success	Alpha	Beta
Failure	Could not be found	Gamma

Additionally, for each case we applied the stakeholder theory (Mitchell et al., 1997) by selecting a sample of all relevant organizations and choosing our interview partners with respect to a rather equal distribution within actor groups and hierarchical levels. Information on potential interviewees were retrieved from the initial interview of each case with the CEO of the sponsor of each platform and documents provided during these interviews. We distinguished between adopters and non-adopters, supporters and non-supporters as well as company sizes. For the individuals from a respective organization, we distinguished between middle or upper management (e.g., director, CEO) and lower management or clerks. By this selection procedure, we could achieve a broad spectrum of opinions and avoid interview biases. To address the known pro-adopter bias from innovation adoption studies (Rogers, 1995) and as all our platform cases are in an early stage, we included both early adopters and non- adopters in our research design already.

The interview guidelines contain semi-structured questions and are categorized into two theme blocks: First, the description of the as-is situation of the platform eco-system and related standards, second, the evaluation and classification of platform eco-system related standards including facilitators and barriers of development and implementation. To ensure a common understanding of our guidelines, we verified them iteratively in several pretests with researchers and industry experts not involved in the design phase.

In total, we conducted 19 interviews (see Table 2) from November 2020 to April 2021. We recorded, transcribed, anonymized the interviews that were held in either German or English based on the preference of the interviewee. German transcripts were translated by the first author and checked for errors independently by two non-involved native English speakers that are proficient in the German language. For the transcription and analysis we used MaxQDA2020 which is a widely used research tool for qualitative research that requires systematic coding procedures (Kuckartz, 2014; Kuckartz & Rädiker, 2019). Each interview was analyzed directly after it was finished to guide the next interview. We terminated the interviews per case after no additional codes were needed and all statements in the transcript could be assigned to the prior developed coding (Eisenhardt, 1989; Wallbach et al., 2019). This was achieved after 13 interviews for all cases because in the fourteenth (*Alpha*), fifteenth (*Beta*+*Gamma*) and sixteenth (*Gamma*) interview no further codes were added. To confirm the termination criterion, we conducted an additional interview per case, which led to the total of 19 interviews. We followed the guidelines of specificity, flexibility, non-direction and range during the interviews (Flick, 2018) and paid attention to a neutral and a nonjudgmental form of listening (Patton, 1990; Walsham, 1995).

**Table 2:** Case and interview metadata; Additional interviews (17 – 19) are marked with an asterisk in the interview duration column

Case (anonymized)	Brief description	Interview duration (mm:ss)	Relation to the respective platform				Small enterprise (< 50 empl.)	Stakeholder (Role)
			Sponsor	Adopter	Supporter	Neither		
*α*	Alpha is a platform in an early stage that aims to connect a range of already settled digital port eco systems which have years or even decades of experience in developing, running and maintaining a successful platform eco system and where the platform sponsor as well as the participating members all have a long history of developing, implementing and enforcing (international) standards. It has a positive outlook on its own future	88:30	x	x	x		x	Platform sponsor α (CEO)
		60:40		x	x			PCS operator A (Technial project leader)
		53:02		x	x			PCS operator B (Regional sales representative)
		78:07			x		x	PCS operator C [× 2] (CEO + Commercial Director)
		36:10*				x	x	Management Consultancy A (Partner)
β	Beta is a platfonn in early stages that aims to revolutionize the intermodal inland and hinterland container transport in Europe. The platform sponsor is a small start up (< 10 employees) that has prior experience in the transportation sector but neither has prior experience with platform eco systems development, nor any previous experience with standard development and adoption. It has a positive outlook on its own future	55:00	x	x	x		x	Platform sponsor β (CEO)
		60:11		x				Freight forwarder + Freight carrier [Road] A (Technical clerk)
		58:15		x			x	Freight forwarder (Sales Manager)
		56:00				x		Inland terminal operator + Freight carrier [barge] A (Director)
		66:39		x	x		x	Inland terminal landlord (Project Manager)
		45:59*				x		Multimodal operator (Project Manager)
γ	Gamma was a platform in early stages that aimed to revolutionize the intermodal inland and hinterland container transport in Europe. The platform sponsor is a small start up (< 10 employees) that has prior experience in the transportation sector and with platform eco systems development, but no previous experience with standard setting and development. It's operations have been terminated	75:36	x	x	x		x	Platform sponsor γ (CEO)
		50:01		x	x			Freight Carrier [Rail + Road] (CEO)
		31:25				x		Freight forwarder + Freight carrier [Road] B (Dispatch & Transport clerk)
		61:10*				x		Inland terminal operator + Freight carrier [barge] B (retired CEO)
β & γ	These stakeholders are external to both cases (β & γ) but have extensive industry knowledge and are familiar with both cases	56:45				x		Lobbying/interest group A (Regional representative)
		48:16				x	x	Lobbying/interest group B (Senior Project Manager)
		58:03				x		Management Consultancy B (Senior Manager)
		55:53				x		Management Consultancy B (Partner)

For the 19 conducted interviews we used three types of coding: open, axial and selective (Hein et al., 2019; Wallbach et al., 2019). Open coding was conducted word-by-word and followed by axial coding to describe the relationships between codes. The results were constantly compared with already coded slices to derive similarities and differences between the cases. Changes in relationships as well as initial insights were documented through memos (Hein et al., 2019). Finally, we conducted a selective coding to derive overarching themes (Wiesche et al. 2017) that are robust along all three cases describing standardization practices (Urquhart 2012). While we aimed to analyze our data without any pre-defined mindset, we still want to be transparent about our education, research, and work background as it could still have influenced our results. In the recent history of preparing this research and the paper at hand, the authors intensely studied multi-level innovation adoption (Kurnia et al., 2019), platform eco-systems (Bogers et al., 2019; de Reuver et al., 2018; Hein et al., 2020) as well as multi-mode standardization (Wiegmann et al., 2017).

To further increase validity and objectivity, we organized a virtual meeting in June 2021 and conducted a confirmatory focus group with four participants plus the first author who acted as the moderator (Tremblay et al., 2010). The meeting was not in-person due to the active Covid19 pandemic. The four participants were selected based on their research or work focus on platform eco-systems, standardization and the cargo transportation industry (e.g., management consulting background). The meeting took a total of 50 minutes, where the first ten minutes consisted of the introduction and problem description and the remaining 40 minutes were spent reviewing, discussing, and adapting the developed framework (Fig. 7).

### Quantification ratio definition

In order to increase the internal generalizability, i.e., to make sure that our conclusions and interpretations are in fact backed by our qualitative data and therefore characteristic of the chosen setting (Maxwell, 2010), we utilize quantification ratios. The validity of our qualitative interpretations and conclusions highly depends on this internal generalizability to the cases under study. The use of quantitative data can also help to identify and characterize the diversity of a case under study and therefore can help to identify patterns that could either be missed by a purely qualitative analyses or which are simply not apparent from unquantitized qualitative data (Maxwell, 2010, pp. 478–479). Utilizing this approach comes with some disadvantages that the authors as well as the readers must be fully aware of. First, one should not infer that internal generalizability leads to an external generalizability. Our setting and sample could still be unrepresentative and therefore conclusions can be limited to the specific context which shall not be addressed using quantification ratios. Additionally, *“[n]umbers can’t replace the actual description of evidence but can provide a supplementary type of support for the conclusions when it’s impossible to present all of this evidence”* (Maxwell, 2010, p. 480). Through the quantification of qualitative data information richness and context is necessarily lost that might be relevant. Therefore, we do not rely exclusively on the quantification ratios but rather use them to present a supplementary summary of certain qualitative findings. Finally, we do not want the reader to assume that our report is in any way more precise, rigorous or scientific just because we use such quantification methods. As elaborated above, we want to use those ratios to increase internal generalizability on the one hand and use them supplementary to our qualitative analysis only.

First, we define a ratio *TRj* [Theme Ratio] that corresponds to the frequency effect sizes or prevalence measures of (Onwuegbuzie, 2003). We analyzed a total of 930 quotations which also form the basis of our open codes. *TRj*, measures the share of quotations relating to one overarching theme (or level) compared to all quotations that relate to either standardization mode (j=1) or standard adoption (j=2). Therefore, we define the number of theme Quotations (*Tq*_*j*_(*t*_*j*_)), which is the sum of all factor quotations (*f*_*i,j*_(*t*_*j*_)) of the respective theme.

$$\begin{array}{c}{TR}_j:\left\{t,\;f\right\}\rightarrow\left[0,\;1\right]\\j\in\left\{1\left(Mode\;of\;standardization\right),2\left(Standard\;Adoption\right)\right\}\\\begin{array}{c}T_j=\left\{t_j\in\mathbb{N}\vert1\leq t_j\leq4\right\}with\;t_j=Overarching\;theme\\F_j=F\left(t_j\right)=\left\{f_{i,j}\left(t_j\right),i,n\left(t_j\right)\in\mathbb{N}\vert1\leq i\leq n\left(t_j\right)\right\}with\;f_{i,j}\left(t_j\right)\\\begin{array}{c}=No.\;of\;quotations\;of\;factor\;i\;in\;theme\;t_j,n\left(t_j\right)=Number\;of\;factors\;in\;theme\;t_j\\{Tq}_j\left(t_j\right)=\sum\nolimits_{i=1}^{n\left(t_j\right)}\left(f_{i,j}\left(t_j\right)\right){\forall f}_{i,j}\in F_j,j\in\left\{1,2\right\}\\{TR}_j\left(t_j\right)=\frac{{Tq}_j\left(t_j\right)}{\sum_{t=1}^4{Tq}_j\left(t_j\right)}{\forall t}_j\in T_j,j\in\left\{1,2\right\}\end{array}\end{array}\end{array}$$

For the detailed results, we define an equivalent ratio per factor (*FR*_*j*_) that measures the weight of each identified factor within its overarching theme, again separated into quotations that relate to either standardization mode (j = 1) or standard adoption (j = 2).

$$\begin{array}{c}{FR}_{j}:\left\{{t}_{j},i\right\}\to \left[\mathrm{0,1}\right]\\ {FR}_{j}\left({t}_{j},i\right)=\frac{{f}_{i,j}\left({t}_{j}\right)}{{T}_{qj}\left({t}_{j}\right)}{\forall t}_{j}\in {T}_{j},{f}_{i,j}\in {F}_{j},j\in \left\{\mathrm{1,2}\right\}\end{array}$$

During the coding, we recognized two peculiarities. First, each factor can be allocated on a scale from external (uninfluenceable) to internal (influenceable) in a short to medium timeframe. Accordingly, we define a third [influenceability] ratio (*IfR*) which indicates the location of a factor on the spectrum between external and internal. In contrast to *TRj* and *FRj, IfR* rather correspond to the latent effect sizes of Onwuegbuzie (2003), as it is more interpretative than *TRj* and *FRj*. We define *IfR* to be:

$$\begin{array}{c}IfR:\left\{t_1,t_2,i\right\}\rightarrow\left[-1,1\right]\\IfR\left(t_1,t_2,i\right)=\frac{I\left(f_{i,1}\left(t_1\right),f_{i,2}\left(t_2\right)\right)-E\left(f_{i,1}\left(t_1\right),f_{i,2}\left(t_2\right)\right)}{f_{i,j}\left(t_j\right)}{\forall t}_{1,2}\in T_{1,2},f_{i,\left(1,2\right)}\in F_{\left(1,2\right)}with\\\begin{array}{c}I\left(\dots\right)=No.\;of\;quotations\;that\;indicate\;an\;internal\;factor,\\E\left(\dots\right)=No.\;of\;quotations\;that\;indicate\;an\;external\;factor\end{array}\end{array}$$

Quotations that indicate neither an internal nor external factor are not considered in the numerator but are part of the denominator. That means that values of *IfR* that are close to (+ 1) indicate an influenceable factor, values close to (-1) indicate an uninfluenceable factor.

Second, factors show interdependencies amongst each other. Therefore, we deviate from the term “independent variables” (see Fig. 1) commonly used in IS innovation adoption research and refer to them as “explanatory variables” (see Fig. 7). To quantify this result, we define a fourth ratio (*IdR*), which captures the interdependence between explanatory variables, corresponding to the latent effect sizes of (Onwuegbuzie, 2003). Whenever two quotations that relate to two different factors occur together within one answer of an interviewee and are coupled through phrases that indicate a dependency (e.g., “*[factor_1] goes with [factor_2]*”), we count them as a dependent set of quotations. As such statements do not necessarily indicate a unidirectional dependency (e.g., A affects B) we assume all dependencies as bi-directional. The set of all dependent quotations for a given factor combination is represented in the formula by ${f}_{{i}_{1},1}\left({t}_{1}\right)\cap {f}_{{i}_{2},1}\left({t}_{1}\right)\cup \left({f}_{{i}_{1},2}\left({t}_{2}\right)\cap {f}_{{i}_{2},2}\left({t}_{2}\right)\right)$.

**Fig. 3:**
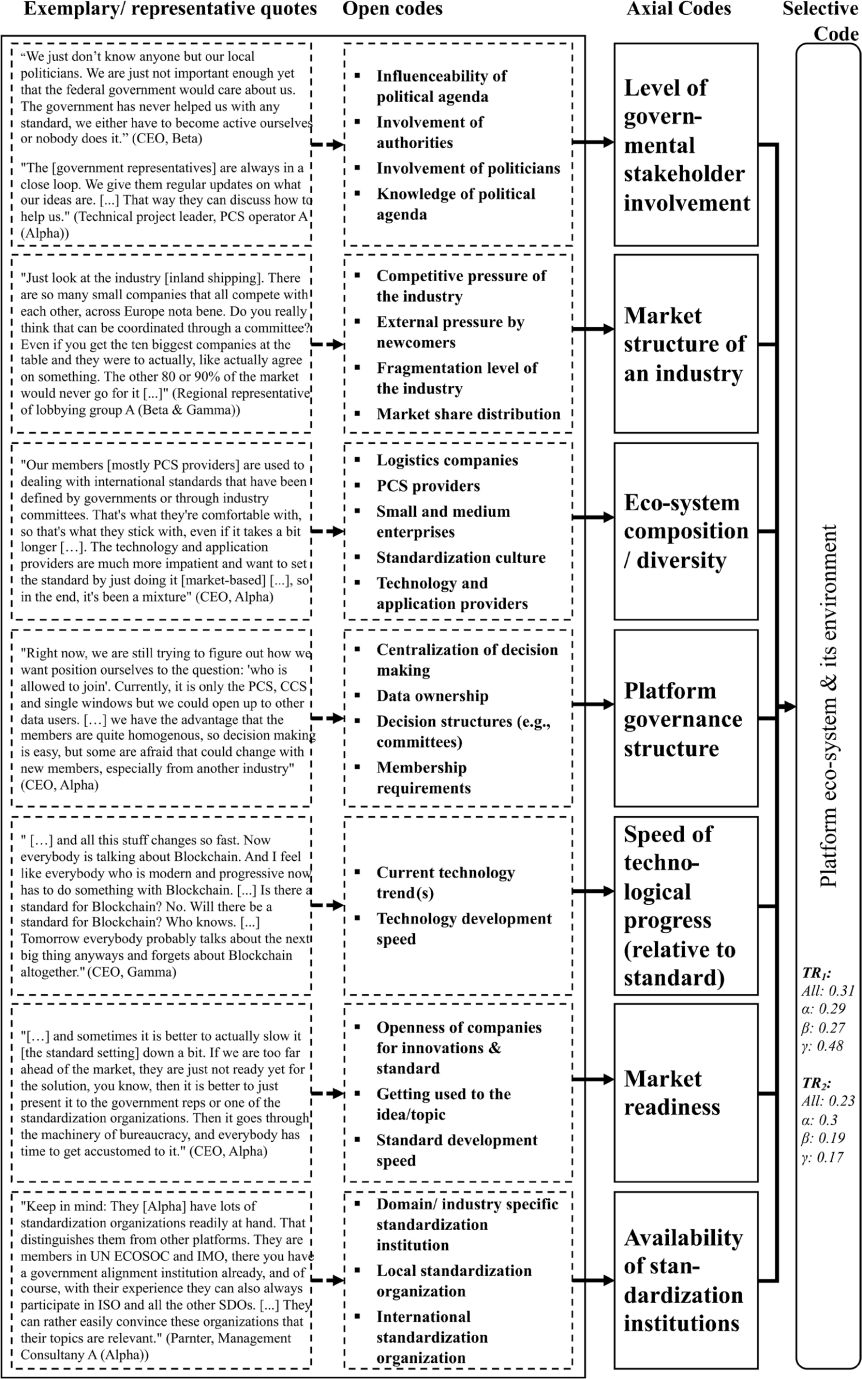
Codes of the Platform eco-system & its environment theme

This set is divided by the smaller total number of quotations of the two factors, as this is equal to the maximum number of quotations in a set.^2^ With this:

$$\begin{array}{c}IdR:\left\{{t}_{1},{t}_{2},{i}_{1},{i}_{2}\right\}\to \left[\mathrm{0,1}\right]\\ IdR\left({t}_{1},{t}_{2},{i}_{1},{i}_{2}\right)=\frac{\left({f}_{{i}_{1},1}\left({t}_{1}\right)\cap {f}_{{i}_{2},1}\left({t}_{1}\right)\right)\cup \left({f}_{{i}_{1},2}\left({t}_{2}\right)\cap {f}_{{i}_{2},2}\left({t}_{2}\right)\right)}{\underset{{i}_{1}{i}_{2}}{\mathrm{min}}\left({f}_{{i}_{1},1}\left({t}_{1}\right)+{f}_{{i}_{1},2}\left({t}_{2}\right),{f}_{{i}_{2},1}\left({t}_{1}\right)+{f}_{{i}_{2},2}\left({t}_{2}\right)\right)}\forall {f}_{i,j}\in {F}_{j},{i}_{1}\ne {i}_{2}\end{array}$$

## Results

### Standard categories in B2B platform eco-systems

The interviews indicate that B2B platform eco-systems are regularly facing and handling a complex set of interacting and interwoven standards, regarding both the core of the platform but even more so for the boundary resources. The *Commercial Director* of a PCS operator involved in *Alpha* descried: “*You always have to consider what’s there already. I think it is better to think of it as a network of standards that all depend on each other*”. Accordingly, we next describe a structuring of the standards relevant for B2B platform eco-systems that emerged from the interviews as it lays a necessary basis for answering RQ1 and RQ2.

The prevalent distinction of standards based on their activation mechanism (mode) is relevant and applicable for our cases also but falls short. A categorization emerged from the interviews that is based upon elements of De Vries (2006)’s typology of IT standards (see Appendix Table 7). We differentiate between two levels of standards, viz. so-called “basic standards” that build the foundation for “requiring standards” (De Vries, 2006). There are basic standards that have an absolute validity such as certain standards for quantities and units (e.g., the International System of Units SI) but there are also other basic standards such as terminology, classification or reference model standards which are context specific. From the context of our cases, an example of a classification standard is the United Nations Code for Trade and Transport Locations (UN/LOCODE), which defines (short) codes for over 100,000 locations in 249 countries (UNECE, 2020). UN/LOCODE is also an example that shows the mutual dependency of standards, as it depends on another basic standard, viz. ISO 3116-1:2020 (ISO, 2020), which defines the codes for the representation of countries. These basic standards have a much longer equilibrium phase compared to requiring standards, i.e., they are used longer without changes.

Requiring standards are defined based on specific requirements which can either be performance-based or design-based (De Vries, 2006). These are usually defined based on a set of basic standards (Tassey, 2000). Take, for example, the dimension standardization of the 20 ft International Standardization Organization (ISO) container in the freight transport sector which enabled an efficient multi-modal transport on sea, rail, road and barge (Egyedi & Spirco, 2011). Without basic standards defining distances (ft and meter) and how to convert those into each other (Panda & Harne, 2014), the standardization process as well as the standard itself could have developed differently. For our context we found both performance-based and design-based standards to be in use. The interviews further revealed that a differentiation between the standards of the core platform and the boundary resources (BR) standards is necessary. BR standards are then further distinguished based on the entities that they connect (see Fig. 7 at the end of this chapter).

### Explanatory variables affecting the mode of standardization and standard adoption

#### Detailed results

We retrieved a total of 24 explanatory variables as axial codes which emerged from a total of 88 open codes derived from the 930 quotations extracted from the 19 interviews. In the following paragraphs we refer to the explanatory variables (axial codes) as “factors”. During the third, selective coding, we aggregated the 24 factors into four distinct levels or overarching themes (selective codes). As we found many factors to affect both the standardization mode as well as the adoption of standards, we present the results for RQ1 and RQ2 jointly.

**Fig. 4:**
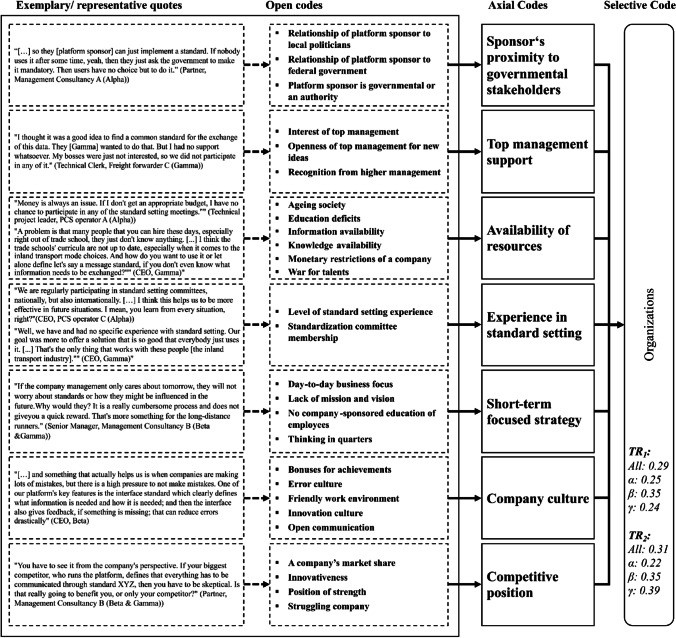
Codes of the organizations theme

**Fig. 5:**
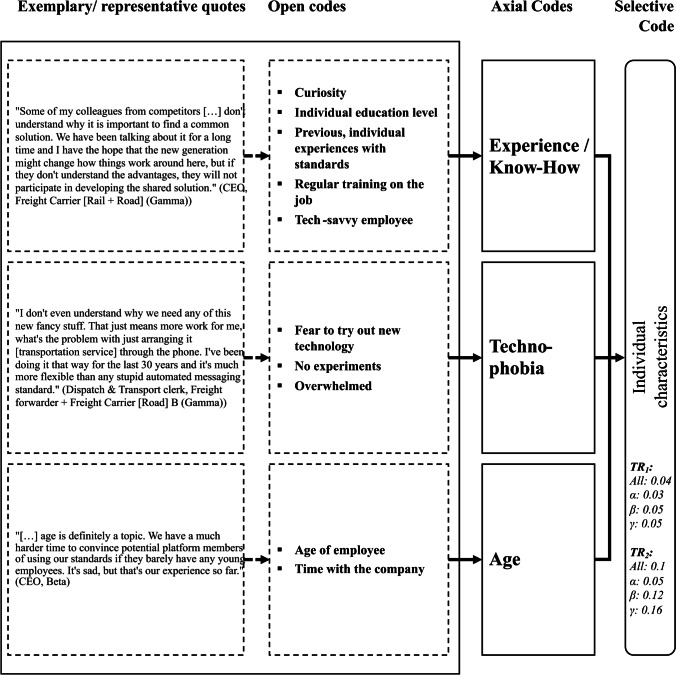
Codes of the individual characteristics theme

**Fig. 6:**
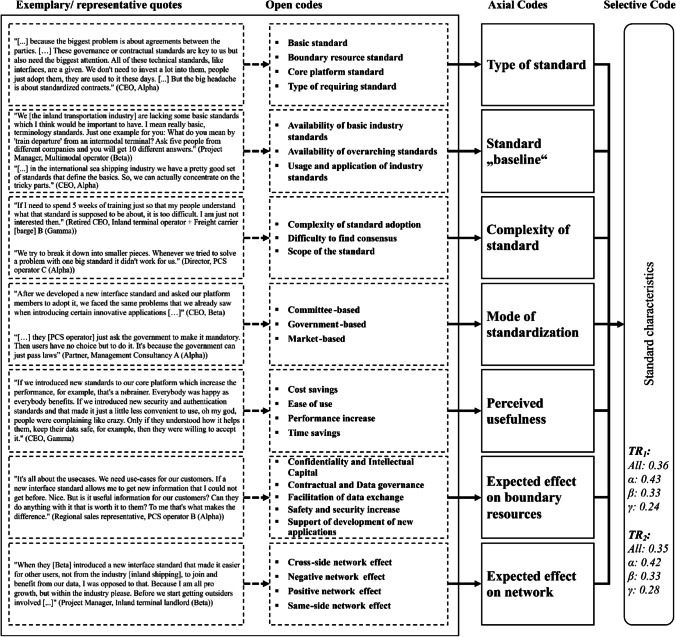
Codes of the standard characteristics theme

Due to space limitations we cannot present all results in detail and have therefore summarized them in one figure and one table per overarching theme (selective code), viz. the platform eco-system and its environment (Fig. 3 and Table 3), characteristics that refer to the involved organizations (Fig. 4 and Table 4) and the individual human (Fig. 5 and Table 5) as well as standard characteristics (Fig. 6 and Table 6). Each figure presents the concept derivation scheme that led to the factors (axial codes) as well as the respective theme (selective code) and lists the aggregated and case-specific TRj values while the respective table presents the explanatory variables (axial codes) per theme (selective code) in more detail by giving a brief description of the variable including the identified effect as well as *FR*_1_ (RQ1) and *FR*_2_ (RQ2), also on a per case basis.

**Table 3:** Overview of explanatory variables from the platform eco-system and its environment

Explanatory variable (Axial code)	Effect on mode of standardization (FR_1_)	Effect on standard adoption (FR_2_)	Brief description
Level of governmental stakeholder involvement	All: 0.18	All: 0.23	The level of governmental involvement in a platform eco-system can vary from external (Beta & Gamma) to deeply involved (Alpha). A deep involvement can ensure that governmental agencies are well-informed about standardization needs, progress and barriers and can accordingly activate the government-based standardization when necessary. Discordant view on the facilitating or impeding role of governmental actors in standard adoption (Alpha: facilitating; Beta & Gamma: impeding). Governments can make standards more attractive, if the usage of a certain standard gurantees compliance with certain regulations
	α: 0.32	α: 0.39	
	β: 0.09	β: 0.08	
	γ: 0.07	γ: 0.05	
Market structure of an industry	All: 0.35	All: 0.31	The market structure of an industry shall describe how competitive and fragmented a market is. By market we refer to all potmtial members of an ecosystem from one industry, whether they already joined the platform or not. We find that the higher levels of competition and fragmentation (e.g., inland shipping industry) make a solely market-based standardization more likely as finding a consensus is more difficult compared to a very concentrated market. The same applies to standard adoption
	α: 0.16	α: 0.15	
	β: 0.51	β: 0.45	
	γ: 0.43	γ: 0.5	
Eco-system composition/diversity	All: 0.11	All: 0.18	The standardization "culture" (Wiegmann et al., 2017) describes the approaches and unwritten conventions regarding the standardization processes of an actor or a group of actors (e.g., industry). On the platform eco-system multiple different actor groups from converging industries are interacting which can have different standardization cultures. We find that a more diverse eco-system that integrates multiple industries with varying cultures can facilitate a multi-mode standardization approach, but the platform sponsor has to be careful that it does not turn into a "clash of cultures;" as this can strongly inhibit the adoption of a standard, especially concerning the boundary resources standards. We find that the eco-system composition also affects the potential of network effects that a standard can generate, which influence the adoption likelihood. An eco-system with more diverse stakeholders has a tendentially higher potential for network effects
	α: 0.12	α: 0.18	
	β: 0.09	β: 0.18	
	γ: 0.13	γ: 0.18	
Platform governance structure	All: 0.2	All: 0.16	The platform governance structure shall describe two levels of openness, complementor openness and decisional openness. We find that if a platform eco-system is only open to developers from one industry for example, it hampers the eco-system diversity and consequently affects the mode of standardization, but can ease the adoption of standards, as the eco-system members are more homogenous. If decisions are made centralized, by one lead-organization platform sponsor, a multi-mode standardization is less likely and it can also hamper the adoption of the resulting standard, as some eco-system members might disagree with the outcomes
	α: 0.24	α: 0.18	
	β: 0.27	β: 0.18	
	γ: 0.03	γ: 0.05	
Speed of technological progress (relative to standard)	All: 0.09	All: 0.04	The speed of technological progress can only affect the standardization mode and standard adoption of those standards that are technology-related (i.e., Machine-Machine and Machine-Human, see Fig. 7). We find that for those standards a fast changing technological environment wi1h high uncertainty tendentially benefits a market-based standardization, as it is seen to be faster and more flexible. Post-hoc legtimization through other modes is seen critical mostly too, as technology has likely developed much further by the time a standard development organization has devdoped a standard. A higher speed of technological progress also has a generally hampering effect on standard adoption, as participants of the platform eco-system are concerned about the longevity of a standard under such circumstances
	α: 0.04	α: 0	
	β: 0	β: 0.03	
	γ: 0.3	γ: 0.18	
Market readiness	All: 0.04	All: 0.08	We find that the market readiness, i.e., the readiness of all potential members of an eco-system from one industry for a standard, whether it is a technical or non-technical standard is a factor that influences the standardization mode. If the market members do not see the benefit ofu new technology (marketbased standardization) at a certain point in time, they will just refuse to adopt it whatsoever and no standard setting is performed. In such cases choosing a committee-based standardization mode can be preferential, where the coordination and accordingly the awareness is created before or during the process development
	α: 0.08	α: 0.1	
	β: 0.02	β: 0.08	
	γ: 0	γ: 0.05	
Availability of standardization institutions	All: 0.03	All: 0	We find that the availability of standardization institutions has a major effect on the mode of standardization as they can enable or ease the activation of committee- and govermnent-based standardization modes. By availability we mean both the sheer existence of such standardization institutions but also the openness and interest of an existing institution in a standardization topic
	α: 0.04		
	β: 0.02		
	γ: 0.03

**Table 4:** Overview of explanatory variables from the individual organizations

Explanatory variable	Effect on mode of standardization (FR_1_)	Effect on standard adoption (FR_2_)	Brief description
Sponsor’s proximity to governmental stakeholders	All: 0.16	All: 0.12	The platform sponsor can have different connections to governmental decision makers (close vs. far). We find that if the platform sponsor is closely related to governmental agencies, the platform eco-system standards can benefit. The government-based standardization mode can be activated much easier at any time then, if seen necessary by the sponsor. This can then affect the standard adoption also, as the government can easier enforce the usage of such standards
	α: 0.38	α: 0.42	
	β: 0.05	β: 0.01	
	γ: 0	γ: 0.02	
Top management support	All: 0.34	All: 0.33	Top management support of both, the platform sponsor and any eco-system member is seen as necessary when it comes to both standard development and adoption and independently from the legal form or other company characteristics in our sample. If the top management of a company or public agency does not see the necessity of a standard, it will not participate in the standard development (committee or government-based mode), will not compete through the market for a standard, but will also not adopt a standard if it was developed already
	α: 0.24	α: 0.22	
	β: 0.41	β: 0.31	
	γ: 0.33	γ: 0.45	
Availability of resources	All: 0.33	All: 0.23	The availability of resources refers to any kind of asset, capability, processes, information, knowledge etc. that a company might have which is necessary or helpful to participate in the standard setting or adoption of a standard. Our interviews show that particularly two types of resources were an issue: Monetary and capability/knowledge related resources. Private as well as public companies cannot participate in any standard setting mode if they lack the monetary capabilites. Similarly, employees that lack the capabilities or knowledge to participate in the development (committee-based) or develop a standard themselves (market-based), are a major barrier for companies to develop a standard. We found the latter to be the case for public agencies also (government-based standardization). Lastly, lacking resources also negatively influence the likelihood of adopting a standard for any platform ecosystem member
	α: 0.24	α: 0.09	
	β: 0.4	β: 0.28	
	γ: 0.33	γ: 0.29	
Experience in standard setting	All: 0.11	All: 0	Any member of the eco-system that is involved in the decision-making (platform governance) can either have previous experience n standard setting or not. We find that previous experience can be an important factor influencing the choice and interaction of standardization modes. When a member has made good experiences in certain standardization modes, it is more likely that it will stick to this standard (standardization "culture" on an organizational level). A higher level of experience also acts as a basis for a more efficient and effective coordination or approach of the market, depending on the mode of standardization
	α: 0.12		
	β: 0.12		
	γ: 0.07		
Short-term focused strategy	All: 0.06	All: 0	Standard setting, independently of the standardization mode, is a comparatively long task for any company or even government:agency. Accordingly, companies that have a short-term focused strategy are less likely participating in standard setting activities
	α: 0.02		
	β: 0.03		
	γ: 0.27		
Company culture	All: 0	All: 0.16	The company culture with all its aspects seems to influence the likelihood with which it will adopt certain standards. On the other hand, we find that a culture that is open to innovation and change tendentially facilitates the adoption of new standards. On the other hand, a culture that does not tolerate errors also seems to facilitate the adoption of standards, especially if the new standard does not replace an old one
		α: 0.18	
		β: 0.2	
		γ: 0.1	
Competitive position	All: 0	All: 0.15	We find that the competitive position of a company affects the standard adoption likelihood. A weaker competitive position in a highly competitive setting seems to hamper the adoption of standards, especially if these standards have been influenced by competitors
		α: 0.09	
		β: 0.2	
		γ: 0.14

**Table 5:** Overview of explanatory variables from individual characteristics

Explanatory variable	Effect on mode of standardization (FR_1_)	Effect on standard adoption (FR_2_)	Brief description
Experience/ Know-How	All: 1	All: 0.55	The involved individual's experience and know-how in the subject-matter is important for both, the standard development as well as ist adoption. If the upper or top management is unknowledgable it can prevent the development within the company (market based) or the participation of the company in a standardization committee or a wrong mode is chosen (e.g., government-based where it does not fit). Unknowledgable employees or employees who are unmotivated due to previous bad experiences can be a barrier to adopting a new standard also
		α: 0.8	
		β: 0.64	
		γ: 0.3	
Technophobia	All: 0	All: 0.25	Technophobia, which is the fear of complex devices or any advanced technology, can act as a major barrier to the adoption of standards. As it is a fear of new technology, it is mostly applicable to technical standards and standards that set rules for the Machine-Human-Interface
		α: 0.1	
		β: 0.16	
		γ: 0.45	
Age	All: 0	All: 0.2	We find that age also plays a role in the adoption likelihood of new standards. Tendentially, a higher age can act as a barrier
		α: 0.1	
		β: 0.2	
		γ: 0.25	

First, for the *platform eco-system and its environment* (Fig. 3 and Table 3) theme we want to present three results that seem particularly noteworthy to us. Generally, interviewees put relatively more emphasis on the effect that the platform eco-system and its environment have on the choice of one or multiple modes of standardization (j = 1) than on the effect that they have on the adoption of a standard (j = 2). Especially the interviewees related to *Gamma* show a strong difference here, while *Alpha’s* interviewees name both effects rather similarly often (Fig. 3). As the Commercial Director of PCS operator C (*Alpha*) puts it: *“Ultimately it [the market structure] affects both. […] We cannot choose a certain way to standardize our interface based on the market and then just neglect it when we try and achieve high adoption rates. It is my core focus to achieve these high adoption rates but I need to also look at how the standard has been defined.”*

Looking more specifically at the factors, two are noteworthy. The *level of governmental stakeholder involvement* is addressed by interviewees of *Alpha* significantly more often than for *Beta* and *Gamma*. The effect that governmental involvement can have on the choice of one or more modes of standardization (j=1) is rather straightforward. As the Partner of Management Consultancy A (*Alpha*) puts it: *“If the government, that is, one of their institutions, such as a ministry or an agency, sees the need for a standard, they can just push that themselves. You know, they either develop it themselves, and you can only hope that someone who knows the topic gets involved in that case, or they pick a standard of their liking”*. More interestingly, there is a discordant view on the effect direction that this factor has on standard adoption (j=2). Most interviewees of *Alpha* see governmental influence as something beneficial to the adoption likelihood of a standard while *Beta* and *Gamma*’s interviewees see this influence to be rather impeding. Exemplarily, this can be seen from the quote excerpt of PCS operator B’s regional sales representative (*Alpha*) who says: *“[…] it was really convenient for me when the government pushed for it [message standard] because all of a sudden it was much easier to “convince” our customers of using the standard. Customs just told them: If you want to comply with our regulations, you better use their system as this is the standard now. Otherwise, there would be penalties, nobody wants to pay penalties”*. Contrarily, the CEO of *Beta*’s platform sponsor states: *“The government has never helped us with any standard, we either have to become active ourselves or nobody does it [de-facto standardization]. And even if they become active, I don’t think it would help much. Whenever any of our customers hear that the government gets involved, they all assume that it will take forever, will be way too complicated and will probably not even work in the end. So, the resistance is really high then, upfront already.”* This shows that a certain perspective bias seems to be present. Platform stakeholders, who (already) experience governmental influence (*Alpha*), are much more aware of how the government can influence the standardization mode and see the effect on the adoption of a standard more positively. On the other hand, stakeholders, who are not used to direct governmental influence (*Beta* and *Gamma*), seem to be less aware of the effect that governmental influence can have or are generally more reserved regarding this influence.

*Beta* and *Gamma*’s interviewees focus much more on the influence that the market structure of the respective industry has than *Alpha*’s. As the regional representative of lobbying group A (*Beta* and *Gamma*) states: *"Just look at the industry [inland shipping]. There are so many small companies that all compete with each other, across Europe nota bene. Do you really think that can be coordinated through a committee? Even if you get the ten biggest companies at the table and they were to actually, like actually agree on something. The other 80 or 90% of the market would never go for it [...]"*. Here, the representative mentions both effects in one quote which other interviewees stated similarly. On the one hand, a highly fragmented market with a competitive environment seems to facilitate a market-based standardization mode, as it is (too) difficult to achieve a coordination through a committee and no market participant is “important” enough to leverage a governmental mode. Thus, fragmentation and competition seem to favor a single mode of standardization as the other two modes are blocked by the circumstances. On the other hand, a highly fragmented and competitive market seems to affect the adoption of a standard, as a standard that would be imposed from the “outside” from a government or a standardization organization could lead to strong resistance and evasion, while a market-based standard is seen as “natural” as the market chose what is best for it, or as the CEO of the freight carrier [Rail + Road] (*Gamma*) puts it: *“It’s just fair that way, isn’t it? We all, as rational businesses, employing business experts, chose the best standard solution. Nobody can tell me what’s best for me, only I can find that out for my business.*

*Learning by doing. I strongly believe that we always find the best solution in the market, over time of course […]*”. If the market is more concentrated, less competitive, and more regulated, such as in *Alpha*’s case, the opposite effect can be seen, i.e., the tendency to use multiple modes and/or specifically not a market-based standardization approach. As the CEO of *Alpha*’s platform states: “*There are just so many PCS providers, you know. It is actually pretty easy. Most of them, if not all, are part in standardization organizations and they all are really closely connected to their local government officials. We just coordinate through those committee, that is clean and lean.”.*

The individual *organizations* (Fig. 4 and Table 4) theme shows relatively high TR values, i.e., its factors have been thematized by interviewees comparatively often. Particularly noteworthy is, that for *Alpha* and *Beta* both the effect on the choice of the standardization mode (j=1) as well as on standard adoption (j=2) have been thematized similarly often. Just for *Gamma*, the failure case in our multiple case study, it differs, as the respective interviewees focus much more on the effect that the individual organizations have on the standard adoption (j=2). As the CEO of *Gamma*’s platform sponsor says: *“It stands and falls with the companies, you know, their culture, the money they have to invest. They cannot or better they wouldn’t adopt our sophisticated, new solution, which I am sure would have become the standard, if they don’t want to or can’t afford it”.* The retired CEO of the inland terminal operator and freight carrier [barge] B (*Gamma*) adds: “[…] *but to me lacking money is not affecting the standardization that much. I mean the way that the standardization is taking place. Because it gets really expensive when you have to implement that. All of these security standards that you have to comply with. A platform can actually help you with that.*” The more successful cases *Alpha* and *Beta* have a more balanced view and see a relatively strong influence of the individual organizations on both, standardization mode(s) and standard adoption.

Analogously to the *level of governmental involvement* factor from the *Platform eco-system and its environment* theme we see for the individual *organizations* theme that interviewees of *Alpha* value the proximity that the platform sponsor, as a singular company, has to governmental stakeholders highly, while *Beta*’s and *Gamma*’s interviewees rarely consider this factor. As the CEO of *Alpha*’s platform sponsor puts it: *“It is really essential to us. Good relationships with all the government people. Even if they are not involved for this project or that project. As we have a close relationship with them, it just helps us to align governmental actions with our needs*”. The technical project leader of PCS operator A (*Alpha*) adds: “*If they [platform sponsor Alpha] wouldn’t have those good relations, I think we would not be where we are today. I mean all of us, we have good relations to our local governments. But they, they really bring it together, cross-border so to say, that’s really important*”. *Beta and Gamma* rather focus on the “classical” IT innovation success factors (see (Jeyaraj et al., 2006)) of *top management support* and *availability of resources*. Four of the identified factors only seem to influence either the mode of standardization (j = 1) or standard adoption (j = 2). While the *experience in standard setting* factor is rather straight forward, we were surprised that the *short-term focused strategy* of a company was only seen as a factor influencing the mode of standardization, but not the adoption of a standard. The CEO of the freight carrier [Rail + Road] (*Gamma*) states: “*The focus is what matters there. Most of the small-scale companies in our industry, they just don’t have the capacity to do lobbying or get themselves involved in some fancy organization committee. They are busy enough getting their everyday work done, it’s all really day-to-day business*”. On the other hand, the *company culture* and *competitive position* of an individual organization seem to only influence the standard adoption in our context. The Senior Project Manager of the lobbying/interest group B (*Beta* & *Gamma*) says: “*The resistance with some market members is really high. But it’s kind of understandable. […] and just assume you are struggling with your business already. If a nice new standard comes around the corner that would force you to change everything, I mean like processes and so on, and that standard was defined by one of your fiercest competitors, I would be hesitant too. Also, many of these small companies, they have been doing their business for 20 or 30 years. I don’t think they have the right innovation mindset*.”

Generally, the *individual characteristics* theme (Fig. 5 and Table 5) has been thematized rarely compared to the other three themes. Nevertheless, especially the interviewees related to the failure case *Gamma* see an influence of these individual characteristics on standard adoption (j = 2) while the effect on the choice of one or more standardization modes (j = 1) has been thematized less.

Both *Alpha* and *Beta* are rather focused on the effect of an individual’s experience and know-how. As the project manager of the inland terminal landlord (*Beta*) states: “*[…] we have this saying: ‘Some people won’t eat anything they’ve never seen before’ and I think that describes it very well. If I don’t know how working in a standardization committee works, I am hesitant to do it, like as an individual you know. And if I, as the CEO, am used to just let the market decide what is the best solution, I will just try to work on that instead of trying some new stuff that I have no experience with”*. The results for the failure case *Gamma* are sticking out, as interviewees pointed out *technophobia* as a major factor influencing the adoption of standards (negatively). *“They are scared, actually afraid of anything new. Not only the management, they sometimes are even open for it. I mean the workers. They’re thrilled to work with a fax. You put paper in, you get a piece of paper out, confirming it was sent. Nice. Most of them don’t even have a smartphone, they still use flip phones. Flip phones. In this day and age […] I think many of them are just getting too old […]”*, describes the CEO of *Gamma*’s platform sponsor on the difficulties they faced when trying to implement a new interface standard for their platform that would not involve fax machines anymore.

The fourth and last theme, viz. *standard characteristics* (Fig. 6 and Table 6), has been thematized relatively often. It was most important to the interviewees of *Alpha*, which is potentially ascribable to the fact that *Alpha “[has] to handle an incredibly wide spectrum of standards. [They] are constantly working on defining, implementing and convincing partner of adopting new and upgraded standards of all sorts*” (CEO of platform sponsor *Alpha*), while both *Beta* and *Gamma* as platforms that are significantly less international and more local from their eco-system focus face a less complex standard and regulation environment.

**Fig. 7:**
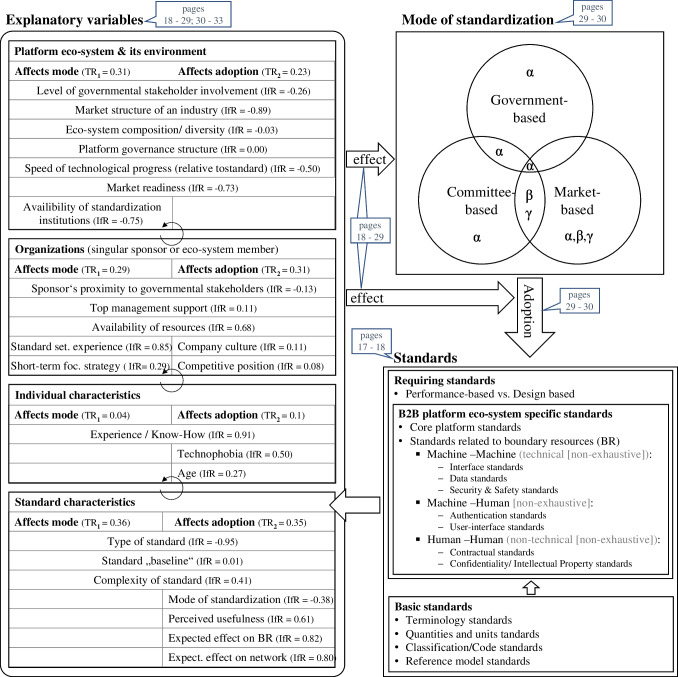
Standard development and adoption framework. Overview of results; “Mode of standardization” box based on (Wiegmann et al., 2017), “Standards” box partially based on results of (De Vries, 2006). α, β and γ in the “Mode of standardization” box refer to the respective cases, for which we found those modes or combination of modes used

We briefly present the results of the standard type, as it is the most thematized factor with an effect on the standardization mode. We find that each type of standard implicates a different mode of standardization or combination of modes which means that a an approach to better standardization needs to consider more than the institutional and technological context (Wiegmann et al., 2017), especially with regards to different standard types. For certain standards (e.g., data standards) it seems advantageous if they are internationally aligned, so a committee-based mode might be advantageous: “*We need that international alignment through ISO. If every country has their own data standard, it is a nightmare. That’s why [Alpha] is so helpful for us*” (Technical project leader – PCS operator A (*Alpha*)). If this alignment is too complicated or takes too long, though (e.g., contractual standards), a combined market- and government-based approach can be preferential, in order to combine the speed of the market-based mode with the hierarchical enforcement power of the government-based mode: “*International committees were not the right place for us to define our contract standards. We just defined it ourselves, I mean all the members aligned, and then we aligned our solution with those agencies for which we knew that they are usually rather strict, Germany for example with regards to data protection clauses. That worked very well for us*” (CEO – platform sponsor *Alpha*). We find that it is advantageous for platform eco- systems in the port context that the international sea transportation industry already has and still is defining an abundance of up-to-date basic standards with a combination of committee-based (e.g., ISO) and government- based (e.g., IMO) modes as this builds a standard “baseline”. Lastly, *Alpha*’s interviewees see more influence of the standard “baseline” on the standardization mode(s) than *Beta* and *Gamma* who thematize the complexity of a standard more often. This seems straightforward, as we find that the standard “baseline” and the complexity of a standard are two dependent factors and that *Alpha*’s standard “baseline” is broader which can reduce the complexity of new standards (see for more information below). Lastly, as *Alpha*’s eco-system has the most experience in multi-mode standardization (see top right of Fig. 7) the interviewees from *Alpha* thematize the mode of standardization as a factor that influences the standard’s adoption significantly more often than *Beta*’s and *Gamma*’s interviewees. So, the mode of standardization is not only a dependent variable (j=1) but is an explanatory variable for the standard adoption (j=2) itself. Interestingly, we find that each mode of standardization has its own effect on the standard adoption (see Table 6). A combination of modes can help to combine certain advantages and disadvantages of individual modes. Standards that follow the market-based mode face similar barriers as (IS) innovations and some of them, such as lacking resources and top management support on a company level might be overcome by also utilizing a government-based mode, as the governmental stakeholders can exert their hierarchical power and push the adoption of a standard. As the partner of Management Consultancy B (*Beta* and *Gamma*) puts it: “*Sometimes I would wish for these new platforms that they would be connected better. They have really good ideas that the entire industry would benefit from, but the market needs a little push, maybe from the government, if nobody else does it*”.

**Table 6:** Overview of explanatory variables from standard characteristics

Explanatory variable	Effect on mode of standardization (FR_1_)	Effect on standard adoption (FR_2_)	Brief description
Type of standard	All: 0.36	All: 0.17	For the type of standard most interviewees focused on the boundary resource (BR) standards of a platform eco-system, which we dinstinguished into machine-machine (technical), machine-human and human–human (non-technical) standards. We find that each of the standard categories seems to have different standardization "cultures" and adoption barriers and faciliators across different member groups of platform eco-systems
	α: 0.38	α: 0.08	
	β: 0.33	β: 0.31	
	γ: 0.33	γ: 0.11	
Standard,,baseline”	All: 0.35	All: 0.1	The standards present in a certain industry context at a given point in time is what we refer to as the "baseline" of standards that the platform eco-system can be built on. We find that a broad and established baseline of standards facilitates the choice of an appropriate standardization mode or a combination of multiple mode and also facilitates the adoption of new standards
	α: 0.45	α: 0.15	
	β: 0.25	β: 0.04	
	γ: 0.27	γ: 0.11	
Complexity of standard	All: 0.29	All: 0.18	The complexity of a standard covers several aspects. It comprises the complexity to find a consensus (controversial standard) the complexity to adopt a standard (incremental vs. revolutionary standard) and the complexity to understand a standard. In summary, we find that the complexity can affect the choice of a standardization mode negatively, as highly complex standards may lead to certain standardization modes not being considered ( e.g., government-based) and also the adoption may be hampered due to long standard development durations
	α: 0.18	α: 0.21	
	β: 0.42	β: 0.1	
	γ: 0.4	γ: 0.26	
Mode of standardization	All: 0	All: 0.14	We find that the mode of standardization affects the likelihood of adoption of certain standards. Government-based standards face less adoption barriers as the government has a high hierarchical power. Committee-based standardizations depend heavily on the committee that develops the standard and if the adopters feel like their needs have been appropriately represented. The market-based standardization mode has many similarities with (other) innovation adoption situations
		α: 0.24	
		β: 0.03	
		γ: 0.09	
Perceived usefulness	All: 0	All: 0.22	The perceived usefulness of a standard is important for its adoption. If potential adopters see it to be useful, they have a much higher likelihood of actually adopting it
		α: 0.14	
		β: 0.29	
		γ: 0.29	
Expected effect on BR	All: 0	All: 0.06	We find that the expected effect of a standard on the boundary resources (BR) affects the likelihood of adoption. The likelihood increases, for example, if the standard is expected to ease the development of new applications or to ease the way of exchanging data, i.e., reduces complexity
		α: 0.11	
		β: 0.01	
		γ: 0.03	
Expected effect on network	All: 0	All: 0.13	Every platform eco-system member has a certain expectation on how a new standard (depending on the type) will affect the network; i.e., the ecosystem. We find that this expectation affects the likelihood of adoption
		α: 0.07	
		β: 0.22	
		γ: 0.11	

#### Standardization and standard adoption as an interconnected, ongoing and dynamic process

As shown in the previous section, many variables influence both the mode of standardization and the standard adoption. We briefly discuss our findings from a viewpoint that does not split up the “decision for one or a combination of modes” and the “standard adoption” but rather see it as one cohesive process. This is in line with the technology management literature’s view on standardization processes which sees the standardization process as comprising three stages, viz. development, selection and implementation (van de Kaa et al., 2011). First, we must distinguish between factors that an individual or a company such as the platform sponsor can influence and others that cannot be easily altered (see *IfR* definition above and Fig. 7 in the following sub-chapter). From the identified factors, most of the organizational and individual characteristics can be influenced. Some of the standard characteristics are influenceable, especially those related to perception and expectations, but also the complexity is addressable by splitting bigger work packages into smaller pieces: “*You really need to make it digestible. If the standard is too complex, it will never be finished, let alone adopted by anyone”* (Senior Manager – Management Consultancy B (*Beta* and *Gamma*)). The standard type, on the other hand, cannot be influenced rather obviously: “*I mean, I cannot change that we have to define a contract standard, it is what it is*” (CEO – Platform sponsor *Alpha*). From the platform eco-system and its environment, some might be influenced easier (platform governance structure) than others (market structure).

Depending on the mode of standardization, the coordination between the standard’s stakeholders takes place at a different time in the process (cf. (Wiegmann et al., 2017)). All identified factors together determine the choice and potential combination of modes as well as the adoption likelihood, but the set of rather external factors must be seen as unalterable during the entire process. A factor combination that leads to one certain combination of modes (e.g., market-based with delayed parallel committee-based standardization followed by a post-hoc legitimization through the government [tri-mode]), does not necessarily lead to a satisfactory adoption of the standard that is developed with these modes: “*We constantly, and when I say constantly, I mean literally every month, adjust where we put our focus. What made you successful yesterday, when you developed something does not necessarily make you successful tomorrow when you have to push it into the market*” (CEO – Platform sponsor *Beta*).

The unswayable factors should be seen as external in the short- and midterm and therefore, companies must focus on those variables that they can influence. Companies must see the standardization process as a “living” process, where boundary conditions change and must actively be changed to achieve better results. An example would be awareness and training campaigns by the platform sponsor during the development process to alter the expectations of the eco-system members regarding the effect of a standard on BR and on the network. Whether this addresses company employees, as it is developed “in-house” [market-based mode], or for a wider group of stakeholders [committee-based mode] is irrelevant.

### Interconnected explanatory variables

We now present those eleven factor-sets with the highest *IdR* values (see supplemental data). First, within the *Platform eco-system and its environment* theme, we find that the *level of governmental stakeholder involvement* is interconnected with the *market structure* of an industry. Both *Beta* and *Gamma* operate in markets (inland/hinterland transport) that are highly competitive and fragmented. In contrast, *Alpha*, is located in a rather monopolistic environment, as its eco-system currently consists only of PCS operators. PCS are monopolistic as there are usually no alternatives for port stakeholders (Mansuri, 2018), although they operate in highly competitive environments (Wallbach et al., 2019). As already indicated in the supply chain context (Farahani et al., 2014), monopolistic markets regularly have regulations imposed by governments and accordingly governmental stakeholders’ interest and involvement in such markets is comparatively high. We find the same in our context and therefore a monopolistic market structure is interconnected with the involvement level of governmental stakeholders.

Furthermore, we find that the platform’s *eco-system composition and diversity* are not independent from its *governance structure*. Especially the complementor openness has a direct effect on the eco-system composition, as it defines who can join the platform under which conditions: “*This openness, it really defines us. We are open to all PCS operators, whoever wants to can join and also influence what we are doing. That is important for our members, quite obviously*” (CEO – Platform sponsor *Alpha*). If the governance structure is rather closed off to complementors, it necessarily constrains the eco-system’s diversity. These governance-related decisions are influenced by the decisional openness of the platform. We find the decisional openness of the platform eco- system to be interconnected to the *market structure*. While a certain *market structure* implies a common decisional governance structure for the network (Farahani et al., 2014; Wallbach et al., 2019), the platform’s governance structure seems to be mirrored. In a monopolistic market, such as *Alpha*’s, the network is lead- organization governed while polypolistic markets, such as *Beta* and *Gamma*, seem to suggest a shared participant governance of the network, as not one single company has the power or influence to set the rules (Wallbach et al., 2019, p. 697). For the platform governance in our cases, we find that the monopolistic market platform *Alpha* has a rather open decisional governance, including many members into decision processes. As *Alpha* horizontally integrates multiple PCS platform eco-systems, one could have expected a more closed-off decisional openness, as these platforms are potentially competing over the same user groups. This is not the case, though, as all platforms that are horizontally integrated here, are locally bounded, i.e., only applied and used in their local (sea)port context. Therefore, they are not competing with each other through *Alpha* but rather cooperating. On the other hand, *Beta* and *Gamma*, which operate in polypolistic networks, apply a more closed- off decisional governance model, where members from the eco-system can make suggestions for changes but the ultimate decision lies solely with the platform sponsor: “*No, our members currently cannot decide where we are going, those decisions lie solely with us*” (CEO – platform sponsor *Beta*).

While the interconnection between the *sponsor’s proximity to governmental stakeholders* and *the level of governmental stakeholder involvement* seems trivial at first, it is not. Governmental stakeholders can be deeply involved in a platform eco-system by setting rules and regulations which does not necessarily entail a close relationship between them and the platform sponsor. Only if the sponsor can align the interests of the platform eco-system with the actions of governmental stakeholders, it is beneficial for the platform and its standardization efforts.

Top management support has been found in many innovation contexts to be a relevant factor influencing adoption (Jeyaraj et al., 2006; Robey et al., 2008) and we find it to affect both standardization mode and standard adoption. It is interconnected with both the availability of resources for a standardization project as well as the three individual characteristics identified. If the general availability of resources is weak, top management support can easily be hampered. Also, if top management support is weak, the availability of resources for the standardization project can be limited: "*I thought it was a good idea to find a common standard for the exchange of this data. They [Gamma] wanted to do that. But I had no support whatsoever. My bosses were just not interested, so we did not participate in any of it. That ultimately meant that I got zero budget for it.*" (Technical Clerk, Freight forwarder C (*Gamma*)). The top management of an organization is influenced by its individual characteristics as much as any employee of any organization involved in the standard setting of platform eco- systems. Accordingly, the individual experience, potentially existing technophobia as well as the age of the top management can influence the support decisions.

As described in Table 3, the speed of technological progress only affects the standardization of technology- related standards, but not standards such as contractual standards which define the interrelation between two non-technical entities. Consequently, an interconnection between the standard type and the speed of technological progress exists. As the Technical project leader of PCS operator A (*Alpha*) puts it: “*Technical progress can be really important depending on the kind of standard we are talking about. Security standards, for example, have to be changed all the time*”. Furthermore, the type of standard is also interconnected with both the existing standard “baseline” and the complexity of a standard. Let us compare two standard type examples from our cases, viz. data standards and contractual standards. *Alpha* and its internationally active PCS operator eco- system members can rely for their data standard(s) on already existing standards (UN/EDIFACT, UN/CEFACT, IMO FAL, IHO S-100, etc.) as well as integration initiatives, such as the recently launched IMO reference data model (cf. (De Cauwer et al., 2021)). Accordingly, *Alpha* has a rich, international standard “baseline” for this standard type. Especially the internationality of the standard “baseline” then leads to a reduced complexity of data standard(s) defined by *Alpha* as it builds solely on existing standards and models. It is important to note that a very rich standard “baseline” can also increase the complexity of a new standard, if various standards are overlapping or contradicting. Therefore, the *“integration initiative of IMO is essential”* (CEO - platform sponsor *Alpha*) for the reduction of complexity in this case. Contrary to *Alpha*, both *Beta* and *Gamma* cannot rely on the same breadth of available data standards for the inland transport sector. For example, different actors in a transport chain have “*[…] different understandings of what a data element ‘Departure from (inland) terminal’ should contain and what the sub-elements mean exactly*” (Director – Inland terminal operator + Freight carrier [barge] A (*Beta*)). In consequence, the standard “baseline” for *Beta* and *Gamma* is so weak, that even basic terminology standards have not yet emerged fully. Accordingly, the complexity of a data standard is much higher for *Beta* and *Gamma* than for *Alpha*, as multiple levels of standards would have to be covered. For contractual standards, the standard “baseline” is again different for each platform. *Alpha* cannot not draw on pre- existing international standards for contractual standards and must define its own standard here. Still, the pre- existing international terminology, classification and data standards build a loosely coupled standard “baseline”, as they are necessary for and thereby facilitate the definition of standardized contractual arrangements between eco-system members. Consequently, from their current position, *Beta* and *Gamma* cannot develop or introduce any contractual standards, as they consider the standard development to be too complex, given their missing standard “baseline”: “*Contractual standards? [Laughs] No we cannot and will not define any contractual standards in the near future. There are much more pressing issues for us that need to be solved first, such as […] data standards*” (CEO – Platform sponsor *Beta*).

### Summary of results

Figure 7 summarizes our main results as presented in the previous sub-chapters. In total we identified 16 variables that affect the choice or interaction of standardization modes (RQ1) and 21 variables that influence on the adoption of a standard (RQ2). It also shows that an overlapping set of factors influences both the standardization mode(s) (j=1) and the outcome of a standard’s adoption (j=2), that the standard characteristics are part of these identified factors, that the factors can change and be actively changed over time so that standardization must be seen as a dynamic process and finally, that the identified factors cannot be considered to be independent from each other. Furthermore, Fig. 7 indicates in the top right, which of three cases experienced which combination of standardization modes and in the bottom right, which standard categorization emerged from our interviews.

Overall, we see from the results that the interviewees of our cases often thematize factors from the “standard characteristics” theme, while “individual characteristics” play a smaller role. The distribution amongst the four themes changes between standardization mode (*TR*_1_) and standard adoption effects (*TR*_2_). Most notably, factors from the “platform eco-system and its environment” theme are more important for the mode choice and combination (*TR*_1_ = 0.31 vs *TR*_2_ = 0.23), while individual characteristics are more important for standard adoption (*TR*_1_ = 0.04 vs *TR*_2_ = 0.1), for example.

## Conclusion

### Concluding discussion of results and implications

Standardization is gaining importance in the platform eco-system context as it is relevant for the acceptance of a comprehensive stakeholder coordination due to the complexity of integrating a variety of stakeholders under a single roof, defining a common ground to work on but also retaining the necessary flexibility for innovations. While platform eco-systems in customer-oriented industries have received abundant research attention in the past, their B2B counterparts have been studied less and almost no research has focused on the standardization dynamics in this environment. Against these backdrops, the objective of our study was to investigate factors influencing the highly dynamic standardization processes of B2B platform eco-systems in a port context. Altogether, we identified 24 factors (explanatory variables) that affect the standardization mode(s) and the adoption of standards, i.e., the dominant paradigm for IT innovation adoption can be applied in the context of standardization. Of those, 16 factors affect the choice or interaction of standardization modes (RQ1) and 21 factors have an effect on the adoption of a standard (RQ2). We categorized the factors into four overarching themes.

On the macro-level, we summarized all factors relating to the platform eco-system and its environment. A distinction of “national”, “industry” and “inter-organizational” levels (Kurnia et al., 2019), for example, seems unpractical in the platform eco-system context, as they are 1) not necessarily bound to one nation (e.g., *Alpha*), 2) multiple converging industries are connected (van de Kaa et al., 2015) and 3) the eco-system is in a constant exchange with its environment also, as new members join while others might leave and therefore the eco-system is not a coherent and stable group of organizations interacting. On an intermediate level, we summarized all factors that relate to the individual member organizations of the platform eco-system and on the micro-level the identified factors concerning the individual employee of an organization. Disparate from those three levels is the last theme, which summarizes factors relating to standard characteristics. These findings indicate that context- specific standard characteristics should be considered in standardization research

Further we find factors affecting standard adoption that are well-known from the innovation adoption field, such as the organizational factors *top management support* and *availability of resources* or the individual factors *experience/ know-how* and *technophobia*. This shows that standard adoption and innovation adoption might be closer related than currently represented by extant research. Somewhat unexpectedly, we did not find *organization size* to be a factor although it is considered highly relevant in the general innovation adoption as well as the PCS adoption context (Jeyaraj et al., 2006; Keceli et al., 2008). We do not preclude its relevance for standardization projects in other contexts, though. Also, somewhat unexpectedly, we find an overlap between factors that influence the mode of standardization and those that influence standard adoption. When viewing the standardization process from a holistic level, involving development, selection and implementation (van de Kaa et al., 2011), the choice of a standardization mode and the adoption of a developed standard are vital parts of this process. All identified factors together determine the choice and potential combination of modes as well as the adoption likelihood, but the set of rather external factors must be seen as unalterable during the entire process. A factor combination that leads to one certain combination of modes (e.g., market-based with delayed parallel committee-based standardization followed by a post-hoc legitimization through the government [tri-mode]), does not necessarily lead to a satisfactory adoption of the standard that is developed with these modes. This shows the need to treat standardization and standard adoption as an interconnected process. The factor that was thematized the most is the *standard type* from the *standard characteristics* theme. Accordingly, we present a framework for distinguishing standards in the B2B platform eco-system context. The *standard type* is interconnected to the *standard “baseline*” that a platform eco-system is facing and the *complexity of the standard* that shall be developed or adopted, which shows another relevant finding of our study. While a major part of the standardization and innovation adoption literature treat factors as linear, independent variables (cf. already (Fichman, 2004)), we find that this is an unfit simplification given the interdependence of many factors. This simplification can ultimately cause endogeneity issues (Guide & Ketokivi, 2015).

Our study contributes to the existing body of research on standardization on the one hand and digital platform eco-systems on the other hand in several important ways. First and foremost, responding to the calls for research from Wiegmann et al. (2017) and Shin et al. (2015), this study is one of the first to systematically and comprehensively investigate factors that influence both the activation of modes of standardization and the standard adoption in a competitive B2B platform eco-system environment from a multi-mode perspective. We can confirm Wiegmann et al., (2017, Chapter 3.3)’s general assumption that standard development is dependent on a standard’s characteristics for the context of platform eco-systems. Also, we find that a close relationship of key platform sponsors to governmental stakeholders is advantageous for activating government-based standardization as they can easier provide relevant information, are aware of the information needs of governments and have causes that the government is intrinsically interested in Wiegmann et al., (2017, Chapter 4.2.2). Finally, we can confirm that standardization has to be seen as an ongoing, dynamic process (Wiegmann et al., 2017, Chapter 4.5). Here we provide additional insights as we allocate the identified factors on a short to midterm influenceability spectrum (*IfR*). Wiegmann et al., (2017, Chapter 4.4.2) mention the transnational standardization of governments and we find for this specific mode of standardization that it is especially useful to agree on certain basic standards that build a baseline for more specific requiring standards which is, to the best of the authors’ knowledge, a novel insight.

Second, we address the calls from IS and IOIS research for multi-level research (Kurnia et al., 2019; Zhang & Gable, 2017) by introducing such multi-level framework to research on standardization processes. First, we take a multi-level view on standards and additionally cluster the identified factors into a multi-level framework.

Although (market-based) standardization and innovation adoption research share many similar concepts (Jeyaraj et al., 2006; Shin et al., 2015), to the best of our knowledge no similarly comprehensive multi-level framework has emerged in standardization research yet. As standardization and standard adoption are becoming increasingly important in platform eco-systems’ business models (Hein et al., 2019; van de Kaa et al., 2015), we also lay the basis for future studies to gain a deeper understanding of sectoral differences in the success factors of digital platform assimilation, as called for by (de Reuver et al., 2018), by providing rich, contextualized insights on factors influencing standardization. Lastly, we add to the limited body of literature on platform eco-systems in B2B contexts (Hein et al., 2019; Loux et al., 2020), with the specialty of strong governmental influence which has not commonly been considered before (Bivona & Cosenz, 2021; de Reuver et al., 2018; Täuscher & Laudien, 2018).

We see a certain similarity between our research and the one performed by Wiegmann (2019) recently. In his case study, Wiegmann (2019) investigates, amongst other things, the management of standards in the European heating sector, specifically the development of the micro Combined Heat and Power (mCHP) technology. He investigates the management of standards in the context of innovations with a link to regulation, which has been mostly neglected before (Wiegmann, 2019). Generally, the “management of standards” can include both our research questions’ aspects, viz. the choice of a standardization mode as well as its adoption and diffusion in the market. Wiegmann (2019)’s focus lies more on the first while it is not completely congruent, as he investigates how a company can manage and potentially influence the standardization process and what that implies for the innovation and new product development processes. While Wiegmann (2019) slightly touches the topic, our study adds the (focused) perspective of what influences the adoption and thereby acceptance of standards. Our results show that it should not be casually assumed that the mere existence of a standard automatically leads to its widespread diffusion, as not all companies are “standard takers” (see (Meyer, 2012)). As a result, Wiegmann (2019) identifies factors on the company and industry level (and beyond) which influence the management of standards. In that sense his approach is similar to ours, as he also chooses a multi-level approach, i.e., does not focus on the company or industry level only. That being said, a different level-split emerges from our grounded theory approach, as we also find the company level (here: “Organizations” theme) and the industry level (here: part of the “Platform eco-system and its environment” theme) to be relevant levels but additionally distinguish an individual human level as well as a standard characteristics level. When looking at the specific results, Wiegmann (2019) finds three factors on the company level to be influencing the management of standardization, viz. the awareness of the importance of standards, the availability of expertise and the availability of (financial) resources. While we find both the availability of expertise and resources to be relevant factors to the choice of one or more standardization modes, we do not directly find the awareness of the importance of standards. Wiegmann (2019) additionally identifies individual knowledge and various organizational measures (see. Chapter 4.1.3. in (Wiegmann, 2019)) to be factors which potentially influence the three aforementioned factors. Especially the individual knowledge factor is interesting here, as this is a key factor of our “Individual characteristics” theme. On an industry level and above, Wiegmann (2019) finds that the supporting institutions, the approach to intellectual property and the backing for innovations in the industry are important factors. While we also identify the availability of standardization institutions to be an important factor influencing the mode of standardization, we did not find the importance of intellectual property for our context. The backing for innovations aspect can, theoretically, be integrated into our “Eco-system composition/diversity” factor, as we found this to influence exactly that, given that different innovation cultures (Wiegmann et al., 2017) are coming together on a (digital) platform. As described above, we identified many more factors influencing both the mode of standardization as well as the adoption of standards. In parts, we ascribe this to the different context that we investigated, as the platforms that we studied bring together many more actors from various industries, compared to the two industries (heating and electrical power generation) that collaborated in Wiegmann (2019)’s cases. Lastly, our fourth theme, i.e., the standard characteristics presents novel insights. In Wiegmann (2019)’s case all standards were of technical nature, i.e., in our categorization either Machine – Machine requiring standards or basic standards. As our cases involved a much wider variety of standards, we show – to the best of the authors’ knowledge – for the first time, which influence different standard characteristics can have.

Lastly, in the transportation research context, we answer the call of A. Moros-Daza et al. (2020) to add to the limited body of holistic research on digital platforms addressing the eco-systems of seaports by studying the standard setting in this context from a multi-mode, multi-level view covering pre- and inter-seaport processes. We also show which factors influence the change of the existing standardization culture through the coalescence of the transportation industry with companies from an IS background and which role governments can play in this. We think that our results should be transferable to other digital B2B platform eco-system contexts as our multiple case study covered a wide range of stakeholders from different industries with varying backgrounds.

Our research provides important insights for practitioners as well. Through the identification of factors and overarching themes influencing standardization, practitioners can develop tailored strategies in order to achieve their standardization goals in platform eco-systems better. Moreover, by evaluating the relevance, influence ability and interdependency of the identified factors, we provide awareness and a prioritization of the factors so that practitioners can better address obstacles during the dynamic standardization process.

### Limitations and future research

As with every study, there remain some limitations to our findings. First, our results might not be transferable to other contexts because it builds on a multiple case study in the digitalized port eco-system context and therefor mostly focuses on stakeholders from cargo transportation, ICT industries and governmental stakeholders. Further studies on platform eco-systems of converging industries that show different mentalities (Wallbach et al., 2019), standardization cultures (Wiegmann et al., 2017), or are in later maturity stages (Tan et al., 2015) can lead to different results. As we applied a multiple case study approach, the well-known limitations of this method apply to our study also (Yin, 2017). In order to address these, we took several steps. First, to increase construct validity, we gathered both primary and secondary data, second, to increase external validity, we applied a replication logic when selecting the cases as we chose them based on polar opposites in key characteristics. Additionally, we selected a sample of all relevant organizations for each case, i.e., did not solely focus on the platform sponsor, as called for by (Hein et al., 2019) and chose our interview partners with respect to equal distribution within actor groups and hierarchical levels. To address the known pro-adopter bias from innovation adoption studies (Rogers, 1995) we included both early adopters and non-adopters in our research design. To further increase validity and objectivity, we also hosted a confirmatory focus group after the analysis of the cases. Lastly, in order to increase the internal generalizability and support our qualitative interpretation (Maxwell, 2010), we define ratios as part of the presentation of our results to quantify our qualitative results (Onwuegbuzie, 2003). Again, one should not infer that internal generalizability leads to an external generalizability, though. Our setting and sample could still be unrepresentative and therefore conclusions can be limited to the specific context which shall not be addressed using quantification ratios. Additionally, through the quantification of qualitative data information richness and context is necessarily lost that might be relevant. Finally, we do not want the reader to assume that our report is in any way more precise, rigorous or scientific just because we use such quantification methods.

Future research can build on our results in many ways. From an IS perspective, researchers could integrate the identified factors into established diffusion theories and frameworks, confirm them in quantitative studies and further develop theories on the interconnectedness of standards and innovations, especially in a platform eco- system context. From a standardization standpoint, several questions remain unanswered, such as “*Which combination of factors leads to the best results?*” or “*Are there certain standard types that are particularly difficult and how should these be addressed?*”. These potential further research questions can be addressed from a multitude of viewpoints and with many different methodological approaches which can also help to overcome the limitations of our study. The first of our two exemplary questions has a certain similarity to (Fichman, 2004)’s concept of “innovation configurations” just in the context of standards and could accordingly be studies with a qualitative comparative analysis approach which can enable researchers to identify the effect of a certain set of factors compared to another set which is especially useful in highly complex contexts such as ours. This could also help to overcome the limitation of our IdR quantification ratio, for which we had to assume that the factor independence is bi-directional and not uni-directional. The second question could be addressed by a longitudinal case study approach, which would allow for deeper insights into specific standardization processes of platform eco-systems which could not be identified by our approach. We hope that our results can build a basis for future research on the topic and fuel the interest in the field.

### Electronic supplementary material

Below is the link to the electronic supplementary material.

Supplementary file1 (XLSX 14 KB)

## Appendix

### Standard typology

Table 7

**Table 7:** Non-exhaustive standard typology aggregated from (De Vries, 2006; Wiegmann et al., 2017)

Subject-matter related	Standard development related	Standard use related
*Matching entities*	*Mode-related*	*Functional*
Thing-to-Thing vs. Man-to-Thing vs. Man-to-Man	Market vs. Governmental vs. Committee	Intrinsic vs. Extrinsic vs. Subjective
*Requirements*	*Geographical reach*	*Business sector*:
Basic standards vs. Requiring Standards (Performance-based vs. Design-based)	Company vs. National vs. Regional vs. International	Engineering, Transport, IT, Housing & Building, …
Interference vs. Compatibility vs. Quality	*Process-related*	*Business model related*
Compatibility: Horizontal vs. Vertical	Anticipatory vs. Concurrent vs. Retrospective	Regulatory vs. Business or Marketing vs. Operational
	Designing vs. Selecting	*Extent of availability*
	Consensus vs. Non-consensus	Public vs. Nonpublic
	Open vs. Closed	Licensed vs. Non-licensed
	Transparent vs. Nontransparent	*Degree of Obligation*
	Visible vs. Invisible Process	Enforced vs. Voluntary
	Stages: Emergent, draft, approval, ...	

### Standardization cultures

With regards to the standardization “culture”, i.e., common practices in standardization of, for example, an industry, the international maritime transportation industry including seaports, shows a different culture compared to the IS domain. The latter is often characterized by “winner-take-all” markets (Schilling, 2002) which lead to fierce battles for (technological) dominance (Suarez, 2004). The result is then a so-called “de- facto” standard (Blind & Gauch, 2008; van de Kaa et al., 2015). This market-based standardization mode is sometimes accompanied by a committee-based mode through consortia or standard developing organizations such as ISO (den Uijl & de Vries, 2013; van den Ende et al., 2012). Contrarily, the international maritime transportation sector is less market competition based and historically focuses more on finding consensuses (de- jure standards), either on a committee or a legal/governmental basis, as the cases of shipping container standardization show exemplarily (Egyedi & Spirco, 2011; Meyer, 2012). The International Maritime Organization (IMO), a United Nation’s agency with currently 174 member states was founded in 1948, is an example for a governmental mode organization. When governments accept IMO conventions, they agree to make it part of their own national law and to enforce it just like any other law (IMO 2021). Both industries with their individual standardization “cultures” converge in our research cases which makes them an interesting study object from a standardization perspective also.

### Horizontally and vertically overlapping platform eco-systems

We propose an additional view on platform eco-system, which incorporates the potential overlapping of two eco- systems, as this is an important concept to distinguish our cases. While a vertical overlapping is rather likely in the platform eco-system context, especially for the consumer-oriented ones, it is rather uncommon horizontally. Let’s take Apple’s app store as an example, which includes many app developers as complementors of the app store’s eco-system (innovation eco-system). Amongst those developers is, for example, Airbnb, i.e., a multi-sided platform company which connects home owners with potential (short term) renters (Guttentag, 2019). With a narrower, innovation-generativity-focused view (Bogers et al., 2019), Airbnb does not meet the characteristics of an eco-system, as it does not foster generativity due to its transactional instead of relational focus (Guttentag, 2019). With a view on eco-systems that integrates transactional supermodularity (Hein et al., 2020), it can be considered an eco-system nevertheless (Hein et al., 2020, pp. 91–92). Following this second viewpoint, the Airbnb eco-system overlaps with Apple’s app store vertically as it offers its app, i.e., one of its access points to the platform, through the app store. It is vertical in the sense that the app store and Airbnb are not competing over consumers, as they are offering two distinct solutions for different problems. What we accordingly call horizontal integration of two platform eco-systems, would be the integration or accessibility of one app store (e.g., Amazon’s) through another app store (e.g., Microsoft’s), as they are potentially addressing the same customer group with a similar value offer (Warren, 2021). The horizontal integration of eco-systems has to be sharply distinguished from a “platform of platforms” concept which would consolidate the offer of a myriad of small, specialized platforms (MacGillivray, 2016). Our understanding of horizontally integrated platform eco- systems differs from this concept as the connection between the two eco-systems is not visible or accessible to an end-user of a respective platform and would just enable this user to access data, services or other artifacts from the connected platform and its respective eco-system.

### Classification of the cases as digital platform eco-systems

Based on the Platform eco-systems chapter above, we present a tabular overview of how the three cases of our case study represent digital platform eco-systems in Table 8 along six characteristics of MSP eco-systems.

Table 8

**Table 8:** Overview of cases as MSP eco-systems

MSP eco-system characteristics\ Cases	Alpha	Beta & Gamma
Complex network of complementors	Connects multiple PCS platforms with their respective members/complementors (including: carriers, terminal operators, trucking companies, freight forwarders, shipping and customs agencies, customs, port authorities, banks, insurances, PCS providers, ICT & tech companies and more)	Connect a wide variety of inland/hinterland transport companies with a focus on continental Europe. Involved complementors include: Terminal operators (sea and inland), Trucking companies, Rail operator, Multimodal operators, Freight forwarders, Carriers, Barge operators, (Inland) port landlords, Customs, Consignor, ICT & tech companies)
Example of:		
Supermodular complementarities	The core supermodular complementarity of all studied platforms is between the availibility of (shipment) information and thequality of services based upon these information. For example, two crucial information for a sea- or inland-port are the estimated arrival time of a vessle (e.g., cmtainer carrier or barge) and shipment goods information (e.g., Bill of lading). Based on these information, other members of the platform can offer services (e.g., availibility of tuck boats, docking availability, refuelling, insurance of goods, etc.). The better the provided information is and the more information is proviled (more information per stakeholders and/or information from more stakeholders), the better get the offered services as they can, for example, be planned better. Assome information (e.g., departure time, latest drop time for containers, etc.) depends on the services provided by a port or a port's stakeholders, this is a ti-directional complementarity	
Paradox of control	The major question here is, how much control shall lie with Alpha and how much with the individual PCS. As each PCS is highly independent and influenced by its local authorities and stakeholders, they require a certain level of independence. On the other hand, a certain standardization and alignment between the various PCS is necessary in order to enable an efficient information exchange	Individual companies involved in the platforms want to have as much control over their information and the information exchange as possible. At the same time, Beta and Gamma both need to make (centralized) decisions in order to make significant progress, also given that they do not have an abundance of resources given their start-up status
Paradox of change	The challenge for Alpha is to provide standardized interfaces so that PCS from all over the world can connect to the platform. On the other hand, the platform and its interfaces needs to be flexible enough to allow for new services and information to be exchanged between the various PCS	The challenge for Beta and Gamma is to provide standardized interfaces so stakeholders from various industries with their proprietary IT systems can connect to the platform and exchange information and receive services. On the other hand, the platform and its interfaces needs to be flexible enough to allow for new services and information to be exchanged between the various stakeholders so that new ideas from the stakeholders can be intergrated without making it impossible for other companies to join later
Digital affordances	One core example of a digital affordance that is offered to the stakeholders of all three platfonns through their respective boundary resources ( e.g. information exchange interface) is the 1:N exchange of information in order to make the shipping process more effective and efficient	
Generativity	Generativity is happening at all three platforms in a very similar way. Take the digital affordance of I:N information excharge. If a company that does not necessarily need a certain information that is exchanged through one of the platforms (e.g. an insurance company does not necessarily need all the shipment-related information to provide a shipment insurance), but can get access to it anyways (e.g., for a fee), it can identify new, tailord services that it can offer with these additional information (e.g., a delay insurance for a shipment, as the insurance company can access the information from multiple sea- and inland-ports easily and estimate the risk of a delay). With the availibility of additional information, such new, unprompted innovations occur on all the studied cases	

#### Case Alpha – Platform for horizontally integrating PCS platforms

Case *Alpha* is a platform that aims at interleaving local PCS amongst each other. In 2021 it is in an early rollout stage after having completed a piloting phase. Its main sponsor is a NGO that has consultative status at the UN ECOSOC (Economic and Social Council) as well as the IMO and the contributors comprise a wide variety of PCS and single window operators. These have various business backgrounds, from governmental agencies, private companies with governmental ownership, public-private partnerships to private companies that have close relations to governmental agencies such as the respective port authority.

The platform mainly aims at facilitating the cross-port, cross-border data exchange, but ultimately a deeper integration of the various local platforms is possible. Local end-users, viz. the various stakeholders of PCS, such as transport network participants, are not directly accessing the platform (horizontally overlapping platform eco- systems). Instead, all transactions are handled by the local PCS that the end-user is signed up with. Therefore, the platform does not require authentication from individual users, as they are identified and verified by their local PCS. Before the initiative, the users of a local PCS could only retrieve information from its respective port community and therefore also only services based on this local information. To utilize information from other ports, they needed to either sign up to these other port’s PCS system or get the information from another involved stakeholder (e.g., carrier). The platform captures its value through license and pay per use fees (which especially cover running costs).

#### Cases Beta and Gamma – Platform eco-systems for the port hinterland and inland cargo transport

The two further platforms with eco-systems are set in the hinterland and inland cargo transport that can but does not have to comprise a seaport (see Fig. 2, both *Beta* and *Gamma* are potentially vertically overlapping with multiple PCS). Both platforms target the highly competitive B2B cargo container transportation market with a European focus (cf. (Jain et al., 2020; Wallbach et al., 2019)). Inland and hinterland transport of containers can generally be organized through three modes (road, rail and barge) and any combination thereof (intermodal, multimodal or combined traffic). While intermodal traffic and a shift from road to other modes of transportation has been in the focus of the European Union and multiple of its member states for decades (UIRR, 2016), the majority of inland and also port hinterland traffic is still relying on road transportation (e.g., (Antwerp Port Authority, 2020)). According to a recent “taxonomy of online exchanges in the freight industry” (Jain et al. 2020), both our cases fall in the category of “freight transport exchanges” (as opposed to “digital freight forwarders”), as they rather aim to facilitate the existing stakeholders instead of replacing them (Jain et al., 2020). To diversify our cases, *Beta* is a platform start-up that is currently in the expansion phase, while *Gamma* is a platform from the same context which has recently been discontinued (failure case).

## Footnotes

1 One has to be aware of the linguistically unfortunate resemblance of the term “(activation) mode of standardization” in the “intermodal” transportation context that we choose. Whenever we mention “mode” or “mutli-mode”, we are set in the context of standardization modes (Wiegmann et al., 2017) and never in a transportation related one (Crainic and Kim, 2007). To avoid confusion, we try to avoid the term “intermodal”.

2 Take, for example, the factor combination (“Top management support” (TMS),”Availibility of resources”(AOR)). In total, i.e., for both effects on the mode of standardization (j = 1) and standard adoption (j = 2), we have ${f}_{TMS,1} + {f}_{TMS,2} = 93$ and ${f}_{AOR,1} + {f}_{AOR,2}= 76$ quotations. This means that we can get maximally 76 dependent sets of quotations, as there are only 76 quotations for AOR. Here, we find 59 dependent sets, which means that $IdR\left(TMS,AOR\right)=\frac{59}{76}\cong 0.78$
